# A Potential Herbal Adjuvant Combined With a Peptide-Based Vaccine Acts Against HPV-Related Tumors Through Enhancing Effector and Memory T-Cell Immune Responses

**DOI:** 10.3389/fimmu.2020.00062

**Published:** 2020-02-20

**Authors:** Ying-Chyi Song, Hui-Chi Huang, Cherry Yin-Yi Chang, Hui-Ju Lee, Chuan-Teng Liu, Hsin-Yi Lo, Tin-Yun Ho, Wu-Chou Lin, Hung-Rong Yen

**Affiliations:** ^1^Research Center for Chinese Herbal Medicine, China Medical University, Taichung, Taiwan; ^2^Chinese Medicine Research Center, China Medical University, Taichung, Taiwan; ^3^Graduate Institute of Integrated Medicine, College of Chinese Medicine, China Medical University, Taichung, Taiwan; ^4^Department of Chinese Pharmaceutical Sciences and Chinese Medicine Resources, College of Chinese Medicine, China Medical University, Taichung, Taiwan; ^5^Department of Obstetrics and Gynecology, China Medical University Hospital, Taichung, Taiwan; ^6^School of Medicine, College of Medicine, China Medical University, Taichung, Taiwan; ^7^Research Center of Traditional Chinese Medicine, Department of Medical Research, China Medical University Hospital, Taichung, Taiwan; ^8^School of Chinese Medicine, College of Chinese Medicine, China Medical University, Taichung, Taiwan; ^9^Department of Chinese Medicine, China Medical University Hospital, Taichung, Taiwan; ^10^Department of Biotechnology, Asia University, Taichung, Taiwan

**Keywords:** cervical cancer, Chinese herbal medicine, *Hedyotis diffusa* Willd, peptide-based cancer vaccine, human papillomavirus

## Abstract

Viral infection is associated with many types of tumorigenesis, including human papillomavirus (HPV)-induced cervical cancer. The induction of a specific T-cell response against virus-infected cells is desired to develop an efficient therapeutic approach for virus-associated cancer. Chinese herbal medicine (CHM) has a long history in the treatment of cancer patients in Asian countries. *Hedyotis diffusa* Willd (Bai Hua She She Cao, BHSSC) is frequently used clinically and has been shown to inhibit tumor growth *in vitro*. However, *in vivo* data demonstrating the antitumor efficacy of BHSSC are still lacking. We showed that BHSSC induces murine and human antigen-presenting cell (APC) activation via the MAPK signaling pathway and enhances antigen presentation in bone marrow-derived dendritic cells (BMDCs) *in vitro*. Furthermore, we identified that treatment with BHSSC leads to improved specific effector and memory T-cell responses *in vivo*. Variant peptide-based vaccines combined with BHSSC improved antitumor activity in preventive, therapeutic, and recurrent HPV-related tumor models. Furthermore, we showed that rutin, one of the ingredients in BHSSC, induces a strong specific immune response against HPV-related tumors *in vivo*. In summary, we demonstrated that BHSSC extract and its active compound, rutin, can be used as adjuvants in peptide-based vaccines to increase immunogenicity and to bypass the requirement of a conditional adjuvant.

## Introduction

Human papillomavirus (HPV) infections may account for the development of several cancers, and a link between HPV infection and squamous cell cancer of the cervix has been identified (1). Cervical cancer, the fourth most common cancer in women, affects women below the age of 45 (2). Nearly all cases of cervical cancer are attributed to infection with high-risk HPV type 16 or type 18. Currently, commercial HPV vaccines contain the HPV capsid protein (L1), which can induce neutralizing antibodies that block HPV type 16/18 viral infection but do not act against virus-infected or cancerous cells (3). Therefore, the development of efficient therapeutic approaches for HPV-associated cancer is still needed.

Moreover, the mechanisms of HPV-induced neoplastic transformation have been determined (4). Oncogenes E6 and E7 are major HPV genes involved in neoplastic transformation, and the main targets of these viral proteins are the cellular tumor suppressor proteins p53 and pRB (5). The HPV E7 oncoprotein is highly conserved among different HPV genotypes (6). Therefore, the E7 protein is suitable for use as a tumor-specific target for therapeutic vaccine development to treat HPV-associated cancer. Due to the emphasis on safety, a new approach for vaccine development involves using highly purified peptides or recombinant proteins as the target antigen; however, these vaccines often have poor immunogenicity.

Chinese herbal medicine (CHM) has a long history in the treatment of various human diseases, including tumors, infections, and autoimmune diseases, in Asian countries. Some studies have demonstrated that CHM may enhance antitumor activity through increasing tumor apoptosis (7–9), inhibiting angiogenesis (10), and modulating the tumor microenvironment (11). In a large-scale clinical analysis, patients with malignant tumors who sought adjunctive CHM treatment had a better overall survival rate than patients who did not undergo CHM treatment (9, 12–14). Among frequently used forms of CHM, *Hedyotis diffusa* Willd (Bai Hua She She Cao, BHSSC) is associated with a reduced hazard ratio for mortality risk in nasopharyngeal carcinoma and breast cancer (9, 12, 14). Moreover, previous investigations have shown that *H. diffusa* Willd possesses important pharmacological activities and is capable of inducing apoptosis in ovarian cancer cells and inhibiting cell proliferation in hepatocellular carcinoma (15, 16). However, the role of *H. diffusa* Willd in the regulation of the immune response and its therapeutic effect on HPV-related cancer have not been investigated.

One of the important issues in cancer immunotherapy is the generation of specific T-cell responses by dendritic cells (DCs). As part of the vaccine development strategy, some weak immunogenic vaccines contain adjuvants to enhance immunity against immunogens and thereby increase the efficacy of the vaccine. Therefore, many various adjuvant strategies for cancer therapy are being examined in preclinical and clinical trials (17). Recently, some studies identified that CHM extracts can be used as adjuvants to induce cellular/humoral immune responses in different vaccine strategies, such as *Clitocybe nuda* extracts, which could induce specific Th1 response in HER-2/neu DNA vaccine; *Carthamus tinctorius* L. extracts, which could enhance cytotoxic T lymphocytes (CTLs) and antitumor response in DC vaccines; *Rhodiola rosea* L. extracts, which could promote cellular and humoral immune responses in OVA protein vaccine; and *Astragalus membranaceus* extracts, which could enhance cellular and humoral immune responses through decreasing the frequency of Tregs in HBV DNA vaccine (18–21). However, none of the abovementioned herbal extracts were used as adjuvants for peptide-based vaccines. As peptide-based vaccination is a vaccine development strategy that enhances efficacy and minimizes toxicity, it would be intriguing to know if CHM can be applied as a safe and effective adjuvant in vaccine generation.

The mechanisms of tumorigenesis in HPV-induced cancer have been reported; however, a practical therapeutic vaccine for HPV-induced cancer is still under development. To develop potentially safer and more effective HPV-related cancer therapies, we screened CHM and showed that *H. diffusa* Willd extract can be used as an immune stimulator combined with a cancer vaccine. Furthermore, this combination endowed vaccines with increased immunologic activity that may be used to bypass the requirement for a conditional adjuvant.

## Materials and Methods

### Cell Lines and Medium

TC-1 was derived from primary lung epithelial cells of C57BL/6 mice. The cells were immortalized with the amphotropic retrovirus vector LXSN16E6E7 (contained HPV16 E6E7 genes) and subsequently transformed with the pVEJB plasmid expressing the activated human c-Ha-ras oncogene, which were a kind gift from Dr. T-C. Wu (Johns Hopkins University, USA) (22). TC-1 cells were cultured in RPMI 1640 medium (Gibco-BRL, NY, USA); the medium was supplemented with 10% heat-inactivated fetal bovine serum (HyClone, Logan, Utah, USA), penicillin (100 U/ml), and streptomycin (100 μg/ml) (Gibco-BRL).

### Peptides, Proteins, and Chemicals

Peptides (Table 1) that contained an MHC class I-restricted CTL epitope derived from the HPV16 E7 protein or the ovalbumin (OVA) protein (23–25) were purchased from GeneDireX (Nevada, USA) or Kelowna International Scientific, Inc. (Taiwan). The purity of all of the peptides was >90%. The peptides were dissolved in dimethyl sulfoxide (DMSO) (Sigma, MO, USA) at a concentration of 10 mg/ml and stored at −80°C until use. OVA protein was purchased from Thermo Fisher (USA) and dissolved in PBS at a concentration of 10 mg/ml. Selected chemicals in *H. diffusa* Willd used in this study (Table S1) were purchased from ChemFaces (Hubei, PRC). The chemicals were dissolved in DMSO at a concentration of 10 mg/ml and stored at 4°C until use.

**Table 1:** Peptides containing MHC class I-restricted CTL epitope derived from HPV16 E7 protein and OVA protein are used in this study.

**Peptide name**	**Original protein**	**Peptide sequence**
RAH (short peptide)	E7 protein	RAHYNIVTF
IDG (long peptide)	E7 protein	IDGPAGQAEPDRAHYNIVTFCCKC
SII (short peptide)	OVA protein	SIINFEKL
EQL (long peptide)	OVA protein	EQLESIINFEKLTEWTSS

*The underline indicates the sequence of MHC class I-restricted CTL epitope*.

### Animals

Female C57BL/6 mice 6–12 weeks of age were obtained from the National Laboratory Animal Breeding and Research Center (Taipei, Taiwan). C57BL/6-Tg (TcraTcrb) 1100Mjb/J mice (the Jackson Laboratory, ME, USA), which are also referred to as OT-1 mice, were designed to recognize OVA peptide residues 257–264 (OVA_257−264_) in the context of H2K^b^ (CD8 coreceptor that interacts with MHC class I). All animals were housed at the Animal Center of China Medical University (CMU) and maintained in accordance with institutional animal care protocol. All of the animal studies were approved by the animal committee of CMU (2017–128).

### Preparation of Chinese Herbal Medicine Extract

*H. diffusa* Willd (BHSSC) powder was obtained from Sun Ten Pharmaceutical Co., Ltd., which is a scientific Chinese medicine. BHSSC powder was dissolved in distilled deionized water for 24 h at room temperature and then with high-pressure saturated steam at 121°C (249°F) for 15 min by an autoclave sterilizer. The insoluble materials were removed by centrifugation at 3,000 rpm for 30 min and stored at 4°C. Extracts were dissolved in water at a concentration of 100 mg/ml and stored at −20°C until use. The method of preparation of CHM extract was described previously (9, 13). The HPLC profile of BHSSC is shown in Figure S1. A stock of this batch of herbal powder is kept in the herbarium in the School of Chinese Medicine, CMU, Taichung.

### Culture of Mouse Bone Marrow-Derived DCs (BMDCs)

BMDCs were harvested as described previously (24, 26). Bone marrow cells from C57BL/6 mice were cultured in Petri dishes at a density of 2 × 10^5^ cells per milliliter in 10 ml of complete RPMI 1640 medium (Gibco, NY, USA) with 20 ng/ml of recombinant mouse granulocyte-macrophage colony-stimulating factor (GM-CSF; PeproTech Inc., New Jersey, USA). Complete RPMI 1640 medium consisted of RPMI 1640 medium supplemented with 10% (v/v) heat-inactivated fetal calf serum, 25 mM HEPES (Biological Industries, Beit HaEmek, Israel), 100 U/ml of penicillin, 100 μg/ml streptomycin sulfate, and 50 μM β-mercaptoethanol (Sigma, MO, USA). On day 3, another 10 ml of complete RPMI 1640 medium containing 20 ng/ml of GM-CSF was added. On day 6, the BMDCs were collected from each dish, washed, and counted. The final population of BMDCs in the culture dish was about 60% (data not shown).

### Culture of Human Monocyte-Derived DCs (MoDCs)

Human MoDCs were harvested as described previously, with minor modifications (27, 28). Human peripheral blood mononuclear cells (PBMCs) were isolated from human blood by Ficoll and Percoll gradients. Monocytes were enriched by adherence as follows: 8 × 10^6^ PBMCs were seeded in 10 cm dishes containing 10 ml of complete RPMI 1640 medium for 18 h, and then the suspended cells were removed by washing with RPMI 1640 medium. After washing, monocytes were cultured in 10 ml of complete RPMI 1640 medium with 100 ng/ml of human GM-CSF (PeproTech Inc., New Jersey, USA) and 100 ng/ml of human IL-4 (PeproTech Inc., New Jersey, USA). On day 3, another 10 ml of complete RPMI 1640 medium containing 100 ng/ml of GM-CSF and IL-4 was added. On day 6, the MoDCs were collected from each dish, washed, and counted. The population of MoDCs in the culture dish was about 30% (data not shown).

### DC Activation and Maturation

To investigate the effect of the CHM extract/compounds on the functional maturation of DCs, 1 × 10^6^ DCs per milliliter were plated in complete RPMI 1640 medium. Then, 100 μg/ml polymyxin B (PMB) (Sigma, MO, USA) with or without CHM extract/compounds was added, and the cells were further incubated for 18 h at 37°C in 5% CO_2_. As a positive control, the cells were incubated with 3 μg/ml of lipopolysaccharide (LPS). PMB has a high affinity for endotoxin and was used to neutralize the effect of endotoxin. Treatment with PMB-spiked LPS was a neutralization control. After incubation, the cells were harvested, and DC surface markers were stained with fluorescence-labeled anti-CD11c, anti-CD40, and anti-CD86 monoclonal antibodies (BD Bioscience, USA). The expression of these markers was analyzed using a FACSVerse instrument (BD Bioscience, San Diego, CA, USA). The supernatants from 18 h cultured cells were isolated and assayed for IL-6 and TNF-α using an ELISA kit (BD Bioscience, USA) according to the manufacturer's protocol.

### Western Blotting

BMDCs (1 × 10^6^/ml) were incubated for 30 min with 100 μg/ml of PMB with or without BHSSC (50, 100, and 150 μg/ml). Whole-cell lysates (15 μg per lane) were separated by 10% sodium dodecyl sulfate–polyacrylamide gel electrophoresis and then analyzed by Western blotting using specific antibodies against ERK/p-ERK, p38/p-p38, JNK/p-JNK, and NFκB/p-NFκB (Cell Signaling Technology, MA, USA). Actin (Merck-Millipore, Darmstadt, Germany) was used as a loading control. ImageJ 1.42q software (NIH Image, National Institutes of Health; online at http://rsbweb.nih.gov/ij/) was used to quantify the data in the image. The density of the actin band was used to normalize differences in loading. Expression levels were compared with those of controls.

### Internalization of DCs

BMDCs were pretreated with BHSSC or rutin (0, 50, 100, or 150 μg/ml) for 18 h at 37°C and then supplemented with 500 μg/ml FITC-labeled dextran (Sigma-Aldrich, MO, USA) for an additional 30 min at 37 or 4°C. The harvested DCs were stained with fluorescence-labeled anti-CD11c Ab (BD Bioscience, USA). The internalization of FITC-labeled dextran by CD11c^+^ cells was analyzed using a FACSVerse flow cytometer. Dead cells were gated out by staining with 1 μg/ml of propidium iodide (PI). After gating on CD11c^+^ cells, the fluorescence intensity of the geometric mean following dextran treatment at 37°C was 100%.

### Presentation of DCs

BMDCs were pretreated with BHSSC or rutin (0–150 μg/ml) for 1 h, and then 50 μg/ml of OVA protein was added for 18 h at 37°C. The harvested DCs were stained with fluorescence-labeled anti-CD11c Ab and anti-mouse H-2Kb bound to SIINFEKL Ab (MHC-SII Ab) (BioLegend, CA, USA). The MHC-SII Ab specifically reacts with the OVA-derived peptide SIINFEKL bound to H-2Kb of MHC class I but not with unbound H-2Kb or H-2Kb bound to an irrelevant peptide. The numbers of antigen-presenting DCs were detected by flow cytometry. The ratio of MHC-SII+CD11c^+^ cells to total cells in the untreated cells was 1. Dead cells were excluded using PI staining.

### Specific CD8^+^ T-Cell Response

OT-1 mice were subcutaneously immunized with PBS, BHSSC (100 μg), OVA (30 μg) mixed with incomplete Freund's adjuvant (IFA), or OVA (30 μg) mixed with BHSSC. After 1 week, the mice were sacrificed, and 1 × 10^6^ splenocytes were incubated with PMA (20 ng/ml) plus ionomycin (1 μg/ml) for 5 h. IFN-γ-secreting cells were stained with fluorescence-labeled anti-CD8 Ab and anti-IFNγ Ab and detected by a FACSVerse flow cytometer.

### *In vivo* Proliferation Assay

C57BL/6 mice were adoptively transferred with 5 μM of CFSE-labeled OT-1 splenocytes (2 × 10^7^). After 24 h, mice were immunized with PBS, 100 μg of BHSSC, 30 μg of OVA long peptide (EQL, Table 1) mixed with IFA, or 30 μg of OVA long peptide (EQL) mixed with 100 μg of BHSSC. On day 3, the mice were sacrificed, and their splenocytes were stained with fluorescence-labeled anti-CD8 Ab. CD8^+^ T-cell proliferation (CD8^+^CFSE1^+^ cells) was analyzed using a FACSVerse flow cytometer.

### Memory T-Cell Response

C57BL/6 mice were subcutaneously immunized with PBS, 200 μg of BHSSC, 30 μg of IDG, or 30 μg of IDG mixed with 200 μg of BHSSC. After 30 days, the immunized mice were sacrificed, and 1 × 10^6^ splenocytes were stimulated with short peptide antigen (RAH, 10 μg/ml) for 4 days. The supernatants from 4 day cultured cells were isolated and assayed for IFNγ using an ELISA kit (BD Bioscience, USA) according to the manufacturer's protocol.

### Tumor Regression Study in Mice

In a preventive model, C57BL/6 mice were single immunized with DMSO, BHSSC (100 μg per mouse), rutin (500 μg per mouse), short peptide antigen (RAH, 10 μg per mouse), or long peptide antigen (IDG, 10 μg per mouse) with or without BHSSC (100 μg per mouse) or rutin (50 or 500 μg per mouse). After 7 days, immunized mice were injected in the abdominal region with 2 × 10^5^ TC-1 cells. The tumor volume and tumor-free survival of the mice were monitored over a 60-day post-tumor implantation period.

In therapeutic model, tumors were first generated by injecting 2 × 10^5^ TC-1 cells into the abdominal region of the mice. At day 6 and 21, tumor-bearing mice were subcutaneously immunized in a separate abdominal region with DMSO, short peptide antigen (RAH, 30 μg per dose), or long peptide antigen (IDG, 30 μg/dose) with or without BHSSC (200 μg per dose), and the tumor volumes were monitored.

In the recurrence model, C57BL/6 mice received a single immunization with 1 μg of IDG per mouse or 1 μg of IDG mixed with 100 μg of BHSSC (IDG + BHSSC) per mouse for 7 days and then implanted with 2 × 10^5^ TC-1 primary tumor cells. At day 40, tumor-free mice were rechallenged with a small number of TC-1 tumor cells (2 × 10^4^). In the other group, tumor-free mice were rechallenged with a large number of TC-1 cells (2 × 10^5^) at day 40. After the rechallenge, the surviving mice were sacrificed at day 60. Their splenocytes were stimulated with RAH for 24 h. After incubation, the cells were harvested. The surface markers on memory T cells were stained with fluorescence-labeled anti-CD8, anti-CD62L, and anti-CD44 monoclonal antibodies (BD Biosciences, USA), and the expression of these markers was analyzed using a FACSVerse instrument (BD Bioscience, San Diego, CA, USA). Furthermore, splenocytes (1 × 10^6^/ml) were stimulated with RAH (10 μg/ml) for 3 days. The supernatant of cultured cells was collected and assayed for IFN-γ (BD Bioscience, USA) using an ELISA kit according to the manufacturer's protocol.

## Results

### The Effects of BHSSC on the Maturation of Antigen-Presenting Cells (APCs)

**Figure 1:**
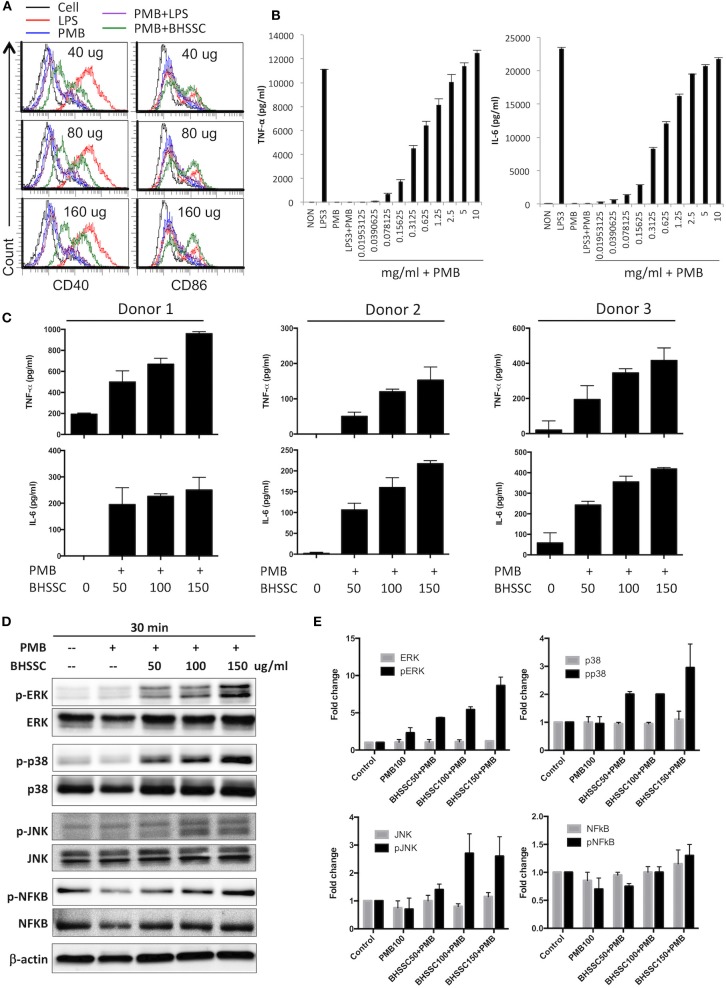
The responses of BMDCs after stimulation with BHSSC. **(A,B)** BMDCs were generated from wild-type C57BL/6 mice and stimulated with the indicated concentration of BHSSC (mg/ml) combined with 100 μg/ml of polymyxin B (PMB) for 18 h. The levels of CD40 and CD86 maturation markers were determined by flow cytometry **(A)**, and the levels of TNF-α and IL-6 in the culture supernatant were measured by ELISA **(B)**. LPS (TLR4 agonist) at a concentration of 3 μg/ml was used as a positive control. PMB has a high affinity for endotoxin and was used to neutralize the effect of endotoxin. **(C)** Human monocyte-derived dendritic cells from three donors were stimulated with different concentrations of BHSSC (0–150 μg/ml) combined with 100 μg/ml of PMB for 18 h. The levels of TNF-α and IL-6 in the culture supernatant were measured by ELISA. The results are expressed as the means + SDs of the level of cytokine. **(D)** BMDCs were stimulated with PMB with or without BHSSC for 30 min. The expression levels of ERK/p-ERK, p38/p-p38, JNK/p-JNK, NFκB/p-NFκB, and actin were determined by Western blotting. **(E)** β-Actin was used to normalize differences in loading. Histograms are presented as fold changes relative to control protein expression level after normalization to β-actin levels. The results are expressed as the means + SEMs. The data shown are representative of two independent experiments.

Some studies have demonstrated that CHM can promote the activation of DCs to regulate immune responses, which are characterized by the increased expression of costimulatory molecules (CD40, CD80, CD86, and MHC II) and several cytokines (IL-1β, IL-6, IL-12p70, and TNF-α) (29, 30). Therefore, we examined the activation of DCs by using water extracts of some individual herbs commonly used to treat cancer patients (Figure S2). We found that only BHSSC and not *Scutellaria barbata* (Ban Zhi Lian) and *Salvia miltiorrhiza* (Dan Shen) could induce TNF-α secretion from BMDCs (Figure S2). Furthermore, we identified costimulatory molecules and other cytokines by treating DCs with BHSSC plus PMB for 18 h. Regarding the effect of BHSSC on the expression of costimulatory molecules, the levels of CD40 and CD86 in mouse BMDCs were enhanced by low-dose BHSSC treatment (Figure 1A). Regarding the secretion of cytokines by mouse BMDCs, the levels of TNF-α and IL-6 were increased via BHSSC treatment in a dose-dependent manner (Figure 1B). In addition, we analyzed adhesion molecules and other costimulatory markers by flow cytometry. These data showed that the levels of costimulatory markers (CD80 and MHC II) and adhesion molecules (integrin β1, integrin α4, c-type lectin, and ICAM-1) were increased by BHSSC treatment (Figure S3). Furthermore, we examined the effect of BHSSC extract on the activation of human MoDCs. Monocytes from three donors were enriched and cultured to induce the formation of MoDCs. The secretion of TNF-α and IL-6 by human MoDCs was increased via BHSSC treatment in a dose-dependent manner (Figure 1C). In addition to DCs, we examined another type of APC, macrophages (RAW264.7 cells), to confirm that BHSSC can also stimulate macrophage activation (Figure S4). Some CHM extracts have been shown to regulate cytokine expression through the NF-κB and MAPK signaling pathways (31–33). We further demonstrated that the level of ERK, p38, and JNK phosphorylation in mouse BMDCs was enhanced in a dose-dependent manner by BHSSC treatment for 30 min (Figures 1D,E). Taken together, these results suggest that BHSSC extract promotes the activation of APCs through the MAPK signaling pathway.

### The Effect of BHSSC on Endocytosis and Antigen Presentation in BMDCs

**Figure 2:**
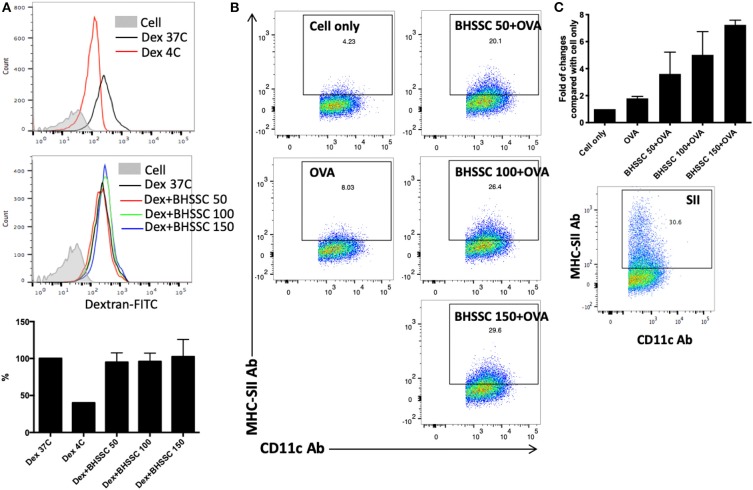
The effect of BHSSC on endocytosis and antigen presentation in BMDCs. **(A)** BMDCs were pretreated with BHSSC (0, 50, 100, or 150 μg/ml) for 18 h and then supplemented with 500 μg/ml FITC-labeled dextran for an additional 30 min at 37 or 4°C (Dex 37C or Dex 4C). Endocytosis was measured by flow cytometry. Bar chart indicating the mean endocytosis percentage. After gating on CD11c^+^ cells, the geometric mean of the fluorescence intensity following dextran treatment at 37°C was 100%. **(B)** BMDCs were untreated (Cell) or pretreated with BHSSC (0, 50, 100, or 150 μg/ml) for 1 h, and then 50 μg/ml of OVA protein was added for 18 h at 37°C. Specific APCs were stained with anti-mouse H-2Kb bound to SIINFEKL Ab (MHC-SII Ab), and their number was detected by flow cytometry. The number in each panel shows the percentage of MHC-SII^+^CD11c^+^ cells in CD11c^+^ cells (specific APCs). **(C)** Bar chart indicating the fold of changes in each group to the untreated (Cell only) group. Dead cells were excluded using PI staining. The lower panel shows the percentage of specific APCs determined by positive control peptide (SIINFEKL) treatment. The data shown are representative of two independent experiments.

DCs are professional APCs in naïve T cells that play an important role in the generation of specific T-cell responses in cancer immunotherapy. Mature DCs are characterized by increased costimulatory molecules (CD40, CD80, CD86, and MHC molecules) and several proinflammatory cytokines (IL-1β, IL-6, IL-12, and TNF-α) and present processed antigens to naïve T cells to promote immune responses (34). Therefore, before identifying the level of specific T-cell responses, we investigated the effect of BHSSC on the functions of DCs, such as endocytosis and specific antigen presentation. To determine the capacity of BMDCs to capture exogenous antigens after BHSSC treatment, the uptake of FITC-labeled dextran by BMDCs was examined by flow cytometry. As shown in Figure 2A, the internalization of FITC-labeled dextran in BMDCs was not significantly different after treatment with various concentrations of BHSSC. To determine the ability of BMDCs to present processed antigens following BHSSC treatment, we determined the number of specific APCs stained with anti-mouse H-2Kb bound to SIINFEKL Ab after OVA antigen treatment. Figure 2B showed us that BHSSC treatment enhanced percentage of specific APCs by BMDCs in a dose-dependent manner (8.0, 20.1, 26.4, and 29.6% for 0, 50, 100, and 150 μg, respectively). The folds of changes in each group to the untreated (cell only) group were 1.8-, 3.6-, 5.0-, and 7.2-fold for 0, 50, 100, and 150 μg of BHSSC treatment, respectively (Figure 2C). These data demonstrate that BHSSC can enhance antigen presentation in BMDCs, but it has no significant effect on the endocytosis of exogenous antigens.

### The Effect of BHSSC on Specific T-Cell Responses

**Figure 3:**
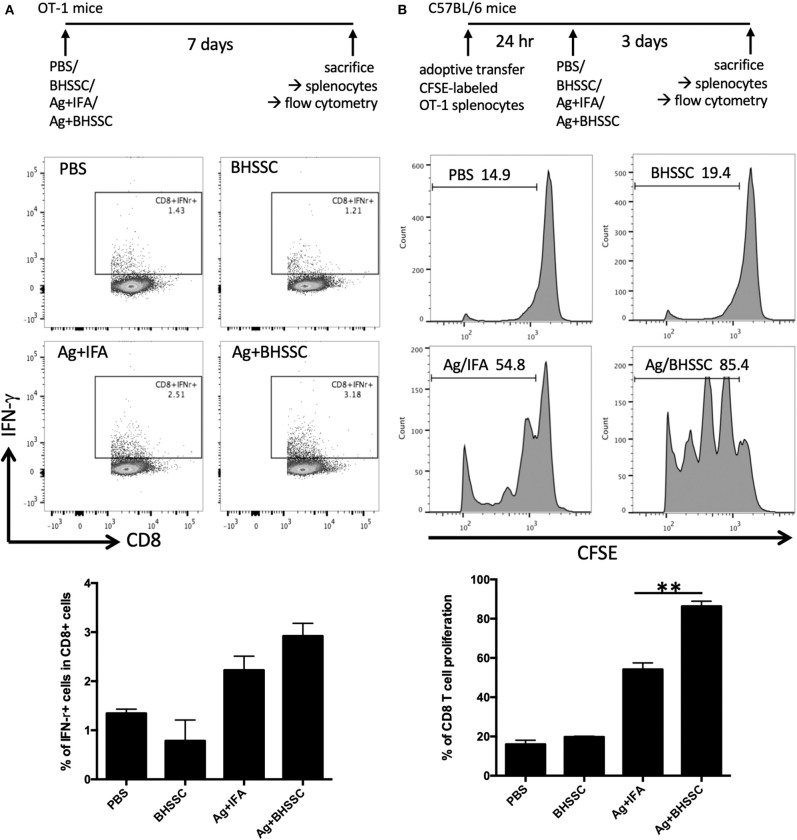
The effect of BHSSC on specific T-cell responses *in vivo*. **(A)** OT-1 mice (*n* = 2 per group) were subcutaneously administered PBS, BHSSC (100 μg), OVA (30 μg) mixed with IFA (Ag + IFA), or OVA (30 μg) mixed with BHSSC (Ag + BHSSC). On day 7, the mice were sacrificed, and their splenocytes were stimulated with PMA (20 ng/ml) plus ionomycin (1 μg/ml) for 5 h. Specific T cells (CD8^+^IFN-γ^+^ cells) were analyzed by flow cytometry. In the panel bottom in **(A)**, the results are expressed as the mean ± SD of the percentage of CD8^+^IFN-γ^+^ cells. **(B)** C57BL/6 mice (*n* = 3/group) were transfected with CFSE-labeled OT-1 splenocytes. After 24 h, the mice were immunized with PBS, BHSSC (100 μg), OVA peptide (30 μg) mixed with IFA (Ag + IFA), or OVA peptide (30 μg) mixed with BHSSC (Ag + BHSSC). After 3 days, the mice were sacrificed, and CD8^+^ T-cell proliferation (CD8^+^CFSE^+^ cells) was analyzed by flow cytometry. In the bottom panel in **(B)**, the results are expressed as the mean ± SD of the percentage of proliferating CD8 T cells. **P < 0.01. The data shown are representative of two independent experiments.

Adjuvants are substances administered along with an antigen to enhance the humoral and/or cell-mediated immune response to the antigen. In the past, antigens were incorporated onto conventional adjuvants (e.g., alum) to gain access to the MHC II pathway for CD4^+^ T-cell activation, but this strategy fails to give antigens access to MHC I processing. Thus, such vaccines fail to stimulate CD8^+^ T cells. Therefore, we investigated the level of the antigen-specific immune response after DC activation following BHSSC treatment. We detected the antigen-specific CD8 T-cell response in OT-1 mice that were designed to recognize ovalbumin peptide residues (OVA_257−264_) in the context of H2K^b^ (CD8 coreceptor that interacts with MHC class I). OT-1 mice were administered PBS, BHSSC, and OVA protein mixed with IFA (Ag + IFA) or BHSSC (Ag + BHSSC). On day 7, their splenocytes were restimulated, and the percentage of specific T cells (CD8^+^IFN-γ^+^ cells) in the Ag + BHSSC group (2.9%) was higher than that in the PBS (1.3%), BHSSC (0.8%), and Ag + IFA (2.2%) groups, as measured by flow cytometry (Figure 3A). To confirm specific CD8 T-cell response *in vitro*, stimulated BMDCs were co-cultured with OT-I CD8 T cells for 3 days. BMDCs stimulated with BHSSC-combined OVA protein induced higher antigen-specific IFN-γ production in OT-I CD8 T cells *in vitro* (Figure S5). Furthermore, to determine the proliferation ability of the specific T cells *in vivo*, wild-type (WT) mice were adoptively transferred with CFSE-labeled OT-1 splenocytes. After 24 h, the mice that received the transferred cells were immunized with PBS, BHSSC, and long peptide antigen mixed with IFA (Ag + IFA) or BHSSC (Ag + BHSSC). On day 3, the proliferation of CD8^+^ T cells (CD8^+^CFSE^+^ cells) was analyzed by flow cytometry. The proliferation ability of CD8 T cells in the Ag + BHSSC group (86%) was greater than that in the PBS (16%), BHSSC (20%), and Ag + IFA (54%) groups (Figure 3B). These data suggest that BHSSC treatment stimulates DC activation (Figure 1), enhances antigen presentation (Figure 2), and promotes DC-induced stimulation of Ag-specific CD8 T-cell immunity (Figure 3).

### BHSSC Extract Combined With Peptides Induced Strong Antitumor Responses

**Figure 4:**
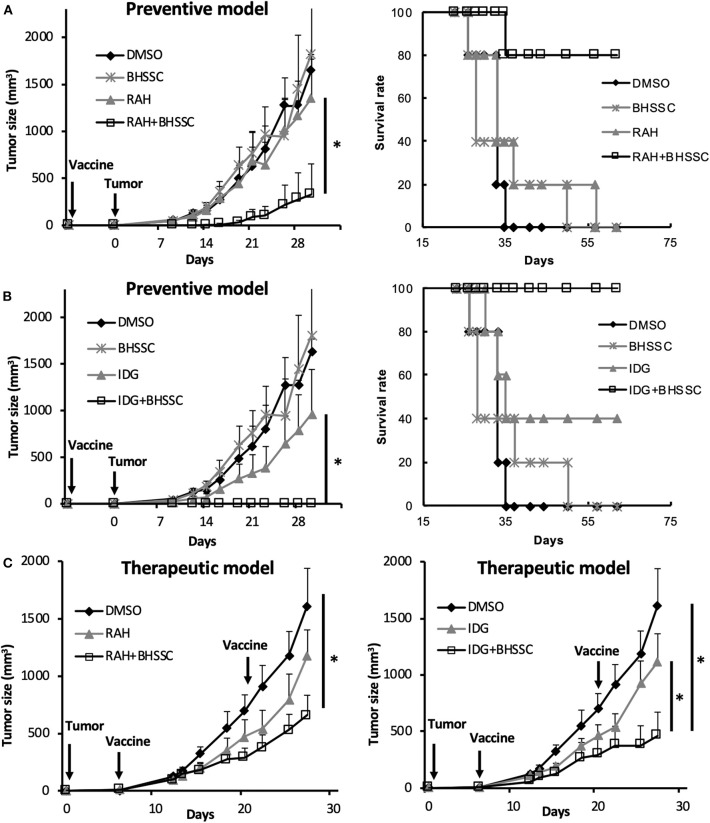
Efficient induction of an antitumor response by the combination of BHSSC with peptide vaccine in preventive and therapeutic models. **(A,B)** C57BL/6 mice (*n* = 5 per group) were injected with DMSO, BHSSC, short peptide antigen (RAH), or long peptide antigen (IDG) with or without BHSSC. In the preventive model, mice received a single immunization with RAH vaccine **(A)** or IDG vaccine **(B)** for 7 days and were then inoculated with TC-1 tumor cells. The left panel indicates the tumor size (mm^3^), and the right panel indicates the tumor-free survival rate (%). **(C)** In the therapeutic model, tumor-bearing mice were immunized with RAH vaccine (left panel) and IDG vaccine (right panel) twice on day 6 and day 21. Tumor diameters were measured with calipers. The results are expressed as the means + SEMs. *P < 0.05. The data shown are representative of two independent experiments.

Cancer vaccines are categorized as either prophylactic or therapeutic interventions. To determine whether BHSSC extract can protect against HPV-associated tumors in a preventive model and a therapeutic model, an HPV E7-expressing TC-1 tumor model was used. In the preventive model, the mice were single injected with different peptide antigens in the presence or absence of BHSSC extract before inoculation with TC-1 cells. As shown in Figure 4A, the mice that were single immunized with short peptide (RAH) plus BHSSC (RAH + BHSSC) displayed significantly delayed tumor growth and had a higher survival rate (80%) than mice in the DMSO, RAH, and BHSSC groups (0%) at day 60. Furthermore, the mice that were single immunized with long peptide (IDG) plus BHSSC (IDG + BHSSC) displayed completely inhibited tumor growth and had a 100% survival rate at day 60 (Figure 4B). However, none of the mice that were immunized with BHSSC alone exhibited inhibited tumor growth. These data suggest that BHSSC might induce antigen-specific immune responses without directly causing cytotoxicity to tumor cells. We have another experiment indirectly indicating that herbal extract containing merely endotoxin would not be strong enough to induce the peptide vaccine's antitumor effects. We chose another Chinese medicine (*Poria cocos* Wolf; Fuling) purchased from the same manufacturer (Sun Ten Pharmaceutical Co., Ltd.) and observed the antitumor effect of a peptide-based vaccine combination under the same extraction conditions and the same *in vivo* experimental procedures. We found that another Chinese medicine extract (Fuling) combined with peptide vaccine (IDG + Fuling) could not enhance antitumor response compared with peptide vaccine (IDG) (Figure S6). We further determined the therapeutic effect of BHSSC. Tumor-bearing mice were injected twice with RAH + BHSSC or IDG + BHSSC vaccine. As shown in Figure 4C, RAH + BHSSC or IDG + BHSSC vaccination had a therapeutic effect and significantly attenuated tumor growth. However, although BHSSC can enhance the antitumor effect of these two peptide vaccines, the IDG + BHSSC vaccine has better prevention and therapeutic effects than the RAH + BHSSC vaccine. As is well-known, cross-presentation by DCs is the major mechanism by which exogenous antigens activate CTLs. Our previous study had demonstrated that long peptides, IDG, could be taken up and cross-presented by DCs but short peptides, RAH, could not. Moreover, long peptides (IDG) had better efficacy to prime specific CD8 T cells than shorter peptides (RAH) (24). Therefore, this study suggested that BHSSC may help long peptide antigens to be presented to CTLs more effectively.

However, tumor cells often induce an immunosuppressive microenvironment that favors the development of immunosuppressive populations of immune cells, such as myeloid-derived suppressor cells (MDSCs), M2 macrophages, regulatory Foxp3^+^ T cells (Tregs), and PD-1^+^ T cells, to escape immune responses. A previous study indicated that some CHM extracts inhibit the MDSC phenotype and exhibit protumor effects (35). To identify the role of BHSSC in PD-1 expression in resting and activated T cells, Jurkat T cells were stimulated with or without anti-CD3 Ab in combination with BHSSC treatment. BHSSC treatment did not modulate PD-1 expression in resting Jurkat T cells and activated T cells (Figure S7A). Furthermore, immunosuppressive cell populations (PD1^+^ CD8 T cells, PD1^+^ CD4 T cells, Tregs, MDSCs, and M2 macrophages) in tumor-bearing mice immunized with RAH + BHSSC or IDG + BHSSC were not significantly altered (Figures S7B–F). On the other hand, the CD8^+^ cells by IDG combined with BHSSC treatment slightly increased (*p* = 0.09) in the tumor microenvironment. However, there were no significant differences in CD4^+^ cells and DCs (Figures S7G–I). Therefore, the combination of peptide antigen and BHSSC elicited superior antitumor activity than that observed without BHSSC treatment but had no effect on immunosuppressive cells in the local tumor microenvironment.

### Expansion of the Memory T-Cell Population

**Figure 5:**
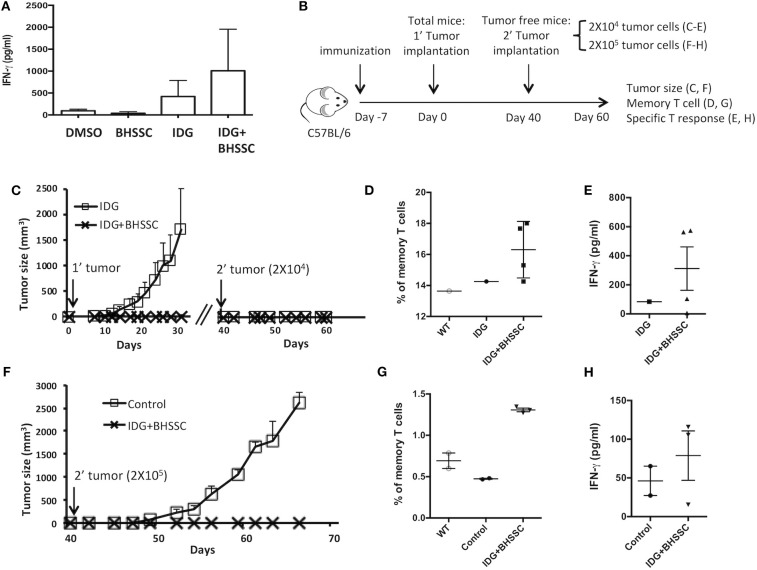
The effect of BHSSC on memory T-cell responses. **(A)** C57BL/6 mice (*n* = 3/group) were subcutaneously immunized with PBS, BHSSC (200 μg), IDG (30 μg), or IDG (30 μg) mixed with BHSSC (IDG + BHSSC). After 30 days, the immunized mice were sacrificed, and their splenocytes were stimulated with RAH (10 μg/ml) for 4 days. The levels of IFN-γ in the culture supernatant were measured by ELISA. **(B)** Experimental timeline. **(C)** C57BL/6 mice (*n* = 10 per group) received a single immunization with IDG or IDG mixed with BHSSC (IDG + BHSSC) for 7 days and were then implanted with TC-1 primary tumor cells. On day 40, the tumor-free mice (*n* = 1 in the IDG group, *n* = 4 in the IDG + BHSSC group) were rechallenged with a small number of TC-1 tumor cells (2 × 10^4^). Tumor diameters were measured with calipers. **(D,E)** On day 60, the surviving mice (*n* = 1 in the IDG group, *n* = 4 in the IDG + BHSSC group) were sacrificed. Their splenocytes were stimulated with RAH for 24 h. Then, the proportion of memory T cells (CD8^+^CD62L^+^CD44^+^) was determined by flow cytometry **(D)**, and the levels of IFN-γ in the culture supernatant were measured by ELISA **(E)**. **(F)** Then, primary tumor-free mice (*n* = 3 in the IDG + BHSSC group) were rechallenged with a large number of TC-1 cells (2 × 10^5^) on day 40. The control group contained naïve mice (*n* = 2) that were challenged with TC-1 cells (2 × 10^5^). **(G**,**H)** The surviving mice were sacrificed at day 60. Their splenocytes were stimulated with RAH for 24 h. Then, the proportion of memory T cells (CD8^+^CD62L^+^CD44^+^) was determined by flow cytometry **(G)**, and the levels of IFN-γ in the culture supernatant were measured by ELISA **(H)**.

Most vaccination programs have focused on inducing cellular immune responses and high antibody titers. However, it is also very important to know if vaccination can induce long-term immunological memory. Therefore, we designed experiments to determine whether a vaccine combining antigen with BHSSC could induce functional memory CD8^+^ T cells. To assess the response of memory T cells, mice were immunized with DMSO, BHSSC, long peptide antigen (IDG), or long peptide antigen combined with BHSSC vaccine (IDG + BHSSC). After 30 days, their splenocytes were stimulated with specific peptide (RAH) (10 μg/ml), and IFN-γ secretion from specific T cells into the culture supernatant was measured by ELISA. The IDG + BHSSC group had a better memory T-cell response than the other groups (Figure 5A). Furthermore, to identify the function of memory T cells in the antitumor response *in vivo*, the immunized mice were rechallenged with TC-1 tumor cells at day 40. Figure 5B shows the experimental timeline. C57BL/6 mice received a single immunization with IDG or IDG mixed with BHSSC (IDG + BHSSC) for 7 days and were then implanted with TC-1 primary tumor cells (Figure 5C). At day 40, tumor-free mice were rechallenged with a small number of TC-1 tumor cells (2 × 10^4^) (Figure 5C) or a large number of TC-1 cells (2 × 10^5^) (Figure 5F). IDG + BHSSC immunization caused the complete inhibition of tumor growth in primary tumor implantation of either a small number of TC-1 tumor cells (Figure 5C) or a large number of TC-1 cells at day 40 (Figure 5F). In the IDG group, the one tumor-free mouse at day 40 also had a memory response to rechallenged tumor cells (Figure 5C). At day 60, splenocytes from the surviving mice were stimulated with RAH, and then the percentage of memory T cells (CD8^+^CD62L^+^CD44^+^) (Figures 5D,G) and the levels of IFN-γ in the culture supernatant (Figures 5E,H) were determined. IDG + BHSSC immunization induced higher levels of memory CD8 T cells and IFN-γ production than those observed in the IDG and control groups (Figures 5D,E,G,H). These results suggest that BHSSC combined with antigen can enhance specific memory T-cell responses and still induce an antitumor response if tumor recurrence occurs.

### Identification of the Active Compounds in BHSSC

**Figure 6:**
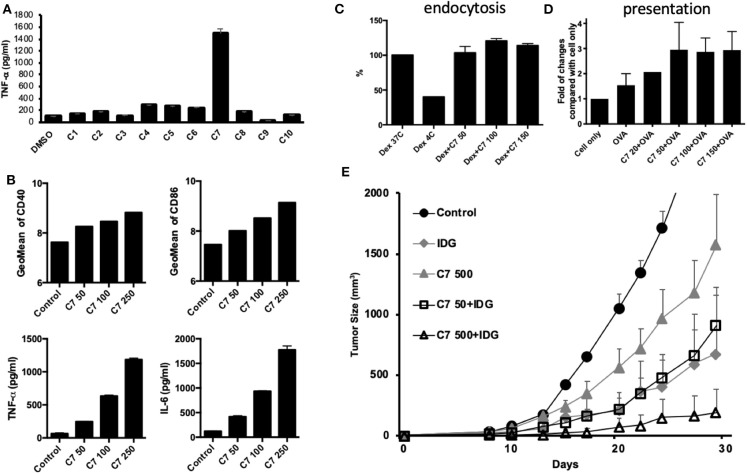
The effects of compounds extracted from BHSSC on BMDC functional activation and their antitumor effects. **(A)** BMDCs were stimulated with different compounds extracted from BHSSC (50 μg/ml) for 18 h. The levels of TNF-α in the culture supernatant were measured by ELISA. **(B)** BMDCs were stimulated with the indicated concentration of rutin (C7, mg/ml) for 18 h. The amount of the CD40 and CD86 maturation markers was analyzed by flow cytometry (top panel), and the levels of TNF-α and IL-6 in the culture supernatant were measured by ELISA (bottom panel). **(C)** BMDCs were pretreated with the indicated concentration of rutin (C7, mg/ml) for 18 h and then supplemented with 500 μg/ml of FITC-labeled dextran for an additional 30 min at 37 or 4°C (Dex 37C or Dex 4C). The effect of rutin on endocytosis was measured by flow cytometry. **(D)** BMDCs were pretreated with rutin (C7, 0–150 μg/ml) for 1 h, and then 50 μg/ml of OVA protein was added for 18 h at 37°C. Specific APCs were stained with anti-mouse H-2Kb bound to SIINFEKL Ab (MHC-SII Ab), and the number of APCs was detected by flow cytometry. **(E)** C57BL/6 mice (*n* = 5/group) received a single immunization with DMSO, 500 μg of rutin per mouse and long peptide antigen (IDG, 10 μg per mouse) with or without rutin (50 or 500 μg per mouse) (C7 50 + IDG/C7 500 + IDG) for 7 days and were then inoculated with TC-1 tumor cells. The results are expressed as the means + SEMs. The data shown are representative of two independent experiments.

According to the results of this study, BHSSC extract may be used as an adjuvant to enhance specific antitumor responses in a preventive or therapeutic HPV-related tumor model. Furthermore, we aimed to identify the active constituent or lead compound in BHSSC extract to facilitate the design of cancer vaccines. A previous study identified some of the constituents of *H. diffusa* Willd (36). On the basis of the chemical profile of *H. diffusa* Willd, we screened 10 compounds from different chemotypes (Table S1). Some of them have been shown to modulate immune responses (37–41). Moreover, all 10 of these compounds can be found in the plasma after intraperitoneal injection of *H. diffusa* Willd water extract (36). We examined BMDC activation by stimulation with these 10 compounds individually. We found that compound 7 (rutin) elicited the greatest DC activation response among all of the compounds (Figure 6A). Moreover, we confirmed that the expression of costimulatory molecules (CD40 and CD86) and cytokines (TNF-α and IL-6) in mouse BMDCs is enhanced by rutin treatment in a dose-dependent manner (Figure 6B). Like treatment with the whole BHSSC extract, rutin treatment enhanced antigen presentation without affecting antigen phagocytosis (Figures 6C,D). Furthermore, we demonstrated that the immunization of mice with peptide (IDG) plus high-dose rutin significantly delayed tumor growth (Figure 6E). Therefore, rutin may be one of the active compounds that induce a specific antitumor response by BHSSC treatment for HPV-related tumors. We also identified rutin in the BHSSC extract by HPLC (Figure S1A). The rutin content in the BHSSC extract was calculated to be 0.048 mg/ml by using HPLC quantification (Figure S1B).

## Discussion

Cancer immunotherapy is a rapidly growing field of cancer research, and cancer vaccines are a promising immunotherapeutic treatment modality. The potential effects of CHM on HPV-related cancer, which are the induction of apoptosis, modulation of gene transcription and protein synthesis, and regulation of immunity, have been demonstrated by *in vitro* and *in vivo* studies (42–45). In this study, we first demonstrated that BHSSC extract can promote the activation of murine and human DCs (Figure 1). Next, we showed that BHSSC enhances antigen presentation in BMDCs (Figure 2), which also results in the stimulation of Ag-specific CD8 T-cell immunity (Figure 3). Furthermore, we demonstrated that a peptide-based vaccine combined with BHSSC extract not only induced a strong antitumor effect in preventive and therapeutic HPV-related tumor models (Figure 4) but also expanded the memory T-cell response in a tumor recurrence model (Figure 5). Additionally, we found that rutin may be one of the active compounds involved in the induction of a specific antitumor response against HPV-related tumors following BHSSC treatment (Figure 6).

In Taiwan, CHMs in the finished product format are manufactured by good manufacturing practice (GMP)-certified pharmaceutical companies (such as Sun Ten Pharmaceutical Co.). These CHM powders (including BHSSC) have fixed production steps and good quality control. Furthermore, we used LAL test (Associates of Cape Cod, Inc. USA) to analyze that 100 μg of BHSSC contained <0.1 μg of endotoxin (data not shown). Therefore, we used 100 μg/ml PMB to neutralize endotoxin in the *in vitro* culture. In addition, we used high doses of LPS (3 μg/ml) to demonstrate that 100 μg/ml PMB can completely inhibit LPS-induced DC maturation (Figure 1B). Additionally, we examined other CHM [*S. barbata* (Ban Zhi Lian) and *S. miltiorrhiza* (Dan Shen)] purchased from the same manufacturer (Sun Ten Pharmaceutical Co., Ltd.) and observed the activation of BMDCs under the same extraction condition and the same *in vitro* experimental procedures (Figure S2). We found that only BHSSC but not *S. barbata* (Ban Zhi Lian) and *S. miltiorrhiza* (Dan Shen) could induce TNF-α secretion from BMDCs. On the other hand, we have an *in vivo* experiment indicating that *P. cocos* Wolf (Fuling) extract from the same manufacturer and extraction condition combined with peptide vaccine (IDG + Fuling) could not enhance antitumor response, compared with peptide vaccine (IDG) (Figure S6). This result confirms that even other herbs containing endotoxins did not influence the peptide vaccine-induced antitumor effect. The BHSSC likely has a better effect compared with other herbal extracts.

Changes in the maturation of DCs may promote Th1 and CTL responses (18, 19, 21, 29). In our study, we found that rutin could activate DCs and enhance antigen presentation to increase antitumor activity in an animal model. Rutin, also called rutoside, is a flavonol glycoside found in many plants, including fruits and vegetables. Rutin is known to have a variety of biological activities, including antioxidant, antiallergenic, anti-inflammatory, and anticarcinogenic properties (46–48). However, the effects of rutin on the regulation of DC function and its application as an adjuvant for cancer vaccine have not yet been investigated. On the basis of the chemical profile of *H. diffusa* Willd, Ye et al. identified rutin in a water extract of *H. diffusa* Willd and plasma after the intraperitoneal injection of *H. diffusa* Willd water extract (36). In this study, we confirmed that rutin is a constituent of BHSSC extracts (Figure S1) and that rutin can activate DCs and enhance antigen presentation to increase antitumor activity in animal models (Figure 6). Based on HPLC quantification, 100 μg of BHSSC contained 2.4 μg of rutin (Figure S1). Even though we found that rutin was one of the active compounds to induce DC activation, increasing the dosage of rutin to 50 μg still did not work better than using 100 μg of BHSSC, which contained only 2.4 μg of rutin (Figure 6). Therefore, maybe there are other effective compounds that work synergistically together with rutin. We will continue to purify and identify the active ingredients in the BHSSC extract in follow-up experiments.

Earlier studies reported the variety of pharmacological effects of BHSSC, including its anticancer, antioxidative, neuroprotective, and immunomodulating activities (49–52). This variety in the effects of BHSSC may be because different components of BHSSC have different effects. Indeed, we found that compound 9 isolated from BHSSC could slightly inhibit cytokine production in DCs (Figure 6A). Conversely, some compounds (compounds 4–7) isolated from BHSSC could also increase cytokine production but not as much as rutin, while other compounds had no effect (Figure 6A). The novelty of our present study is the employment of BHSSC extract as an effective adjuvant in peptide-based vaccines to induce a specific immune response.

Furthermore, some previous studies demonstrated that *H. diffusa* Willd can induce apoptosis in ovarian cancer cells, leading to the inhibition of tumor growth in hepatocellular carcinoma (15, 16). To verify this, we tested the viability of TC-1 cells treated with BHSSC. BHSSC treatment did not induce cytotoxic effects on TC-1 cells even at a concentration of 1,000 μg/ml (Figure S8A). Moreover, we also examined whether BHSSC treatment affects BMDC viability. There was also no cytotoxic effect on BMDCs following BHSSC treatment at all the doses used in this study (Figure S8B). In addition, mice immunized with BHSSC alone did not exhibit tumor growth inhibition (Figures 4A,B). This could be due to the BHSSC extract having different cytotoxic effects on different forms of cancer. We confirmed that treatment with BHSSC water extract in combination with a peptide-based vaccine has a therapeutic effect on HPV-associated cancer resulting from increased specific immune responses but not increased tumor cell apoptosis.

Some CHM extracts/compounds, such as *Pleurotus ferulae* and Korean mistletoe lectin, have been shown to promote the activation of DCs through TLR-mediated signaling pathways (29, 30). Therefore, to determine whether BHSSC activates BMDCs through TLR, we measured the levels of TNF-α and IL-6 in WT and MyD88-deficient (MyD88-KO) BMDCs by ELISA. A TLR4 ligand, LPS, was used as a control. As shown in Figure S9, LPS increased TNF-α production in WT BMDCs and decreased TNF-α production in MyD88-KO BMDCs. However, TNF-α induction by BHSSC + PMB treatment was only partially inhibited in MyD88-KO BMDCs compared with WT BMDCs (Figure S9). Therefore, BHSSC-induced cytokine production in BMDCs may be partially mediated through the TLR signaling pathway. The emerging paradigm of cancer immunotherapy consists of not only the activation of effector T cells but also the reawakening of silenced immune responses (53). Our study demonstrates that BHSSC treatment does not affect PD-1 expression *in vitro* (Figure S7A) or modulate immunosuppressive populations *in vivo* (Figures S7B–F). Therefore, the mechanisms by which BHSSC improves immune responses may be independent of both the stimulation of TLR signaling and the modulation of immunosuppressive factors.

In conclusion, we demonstrated that BHSSC enhances BMDC activation and antigen presentation on BMDCs, induces specific effector and memory T cells, and promotes therapeutic and protective antitumor responses. Furthermore, we determined that rutin, a compound extracted from *H. diffusa* Willd, can induce DC activation and delay tumor growth; rutin is therefore an active compound involved in the induction of the specific antitumor response against HPV-related tumors in BHSSC treatment. Based on this study, the combination of an herbal adjuvant with a peptide-based vaccine may be a novel strategy against virus-associated tumors.

## Data Availability Statement

The datasets generated for this study are available on request to the corresponding author.

## Ethics Statement

The studies involving human participants were reviewed and approved by the Research Ethics Committee of China Medical University and Hospital, Taiwan. The patients/participants provided their written informed consent to participate in this study. The animal study was reviewed and approved by the Animal Committee of China Medical University, Taiwan.

## Author Contributions

Y-CS, W-CL, and H-RY conceptualized the study and drafted the manuscript. Y-CS and H-RY supervised the project. Y-CS, CC, C-TL, W-CL, and H-JL contributed to the *in vitro* and *in vivo* experimental studies. H-CH contributed to the collection and analysis of the HPLC data. H-YL and T-YH prepared the herbal samples. Y-CS and H-RY finalized the manuscript.

### Conflict of Interest

The authors declare that the research was conducted in the absence of any commercial or financial relationships that could be construed as a potential conflict of interest.

## References

[1] zur Hausen H. Condylomata acuminata and human genital cancer. Cancer Res. (1976) 36(2 Pt 2):794. 175942

[2] Ferlay J Soerjomataram I Dikshit R Eser S Mathers C Rebelo M. Cancer incidence and mortality worldwide: sources, methods and major patterns in GLOBOCAN 2012. Int J Cancer. (2015) 136:E359–86. 10.1002/ijc.29210 25220842

[3] Chabeda A Yanez RJR Lamprecht R Meyers AE Rybicki EP Hitzeroth II. Therapeutic vaccines for high-risk HPV-associated diseases. Papillomavirus Res. (2017) 5:46–58. 10.1016/j.pvr.2017.12.006 29277575 PMC5887015

[4] Akagi K Li J Broutian TR Padilla-Nash H Xiao W Jiang B. Genome-wide analysis of HPV integration in human cancers reveals recurrent, focal genomic instability. Genome Res. (2014) 24:185–99. 10.1101/gr.164806.113 24201445 PMC3912410

[5] Tindle RW. Immune evasion in human papillomavirus-associated cervical cancer. Nat Rev Cancer. (2002) 2:59–65. 10.1038/nrc700 11902586

[6] Stanley MA Pett MR Coleman N. HPV: from infection to cancer. Biochem Soc Trans. (2007) 35:1456–60. 10.1042/BST0351456 18031245

[7] Lam W Jiang Z Guan F Huang X Hu R Wang J. PHY906(KD018), an adjuvant based on a 1800-year-old Chinese medicine, enhanced the anti-tumor activity of Sorafenib by changing the tumor microenvironment. Sci Rep. (2015) 5:9384. 10.1038/srep09384 25819872 PMC4377583

[8] Wang Q Li H Sun Z Dong L Gao L Liu C. Kukoamine A inhibits human glioblastoma cell growth and migration through apoptosis induction and epithelial-mesenchymal transition attenuation. Sci Rep. (2016) 6:36543. 10.1038/srep36543 27824118 PMC5099904

[9] Chou ST Hsiang CY Lo HY Huang HF Lai MT Hsieh CL. Exploration of anti-cancer effects and mechanisms of Zuo-Jin-Wan and its alkaloid components *in vitro* and in orthotopic HepG2 xenograft immunocompetent mice. BMC Complement Altern Med. (2017) 17:121. 10.1186/s12906-017-1586-6 28219365 PMC5319192

[10] Yue GG Xie S Lee JK Kwok HF Gao S Nian Y. New potential beneficial effects of actein, a triterpene glycoside isolated from Cimicifuga species, in breast cancer treatment. Sci Rep. (2016) 6:35263. 10.1038/srep35263 27731376 PMC5059658

[11] Yin SY Jian FY Chen YH Chien SC Hsieh MC Hsiao PW Induction of IL-25 secretion from tumour-associated fibroblasts suppresses mammary tumour metastasis. Nat Commun. (2016) 7:11311 10.1038/ncomms11909 27089063 PMC4837478

[12] Lee YW Chen TL Shih YR Tsai CL Chang CC Liang HH. Adjunctive traditional Chinese medicine therapy improves survival in patients with advanced breast cancer: a population-based study. Cancer. (2014) 120:1338–44. 10.1002/cncr.28579 24496917

[13] Song YC Hung KF Liang KL Chiang JH Huang HC Lee HJ. Adjunctive Chinese herbal medicine therapy for nasopharyngeal carcinoma: clinical evidence and experimental validation. Head Neck. (2019) 41:2860–72. 10.1002/hed.25766 30985039

[14] Yeh YC Chen HY Yang SH Lin YH Chiu JH Lin YH. Hedyotis diffusa Combined with Scutellaria barbata are the core treatment of Chinese herbal medicine used for breast cancer patients: a population-based study. Evid Based Complement Alternat Med. (2014) 2014:202378. 10.1155/2014/202378 24734104 PMC3966415

[15] Song YH Jeong SJ Kwon HY Kim B Kim SH Yoo DY. Ursolic acid from Oldenlandia diffusa induces apoptosis via activation of caspases and phosphorylation of glycogen synthase kinase 3 beta in SK-OV-3 ovarian cancer cells. Biol Pharm Bull. (2012) 35:1022–8. 10.1248/bpb.b110660 22791147

[16] Sunwoo YY Lee JH Jung HY Jung YJ Park MS Chung YA. Oldenlandia diffusa promotes antiproliferative and apoptotic effects in a rat hepatocellular carcinoma with liver cirrhosis. Evid Based Complement Alternat Med. (2015) 2015:501508. 10.1155/2015/501508 25852766 PMC4379430

[17] Lin K Doolan K Hung CF Wu TC. Perspectives for preventive and therapeutic HPV vaccines. J Formos Med Assoc. (2010) 109:4–24. 10.1016/S0929-6646(10)60017-4 20123582 PMC2908016

[18] Chen MH Li WS Lue YS Chu CL Pan IH Ko CH. Clitocybe nuda activates dendritic cells and acts as a DNA vaccine adjuvant. Evid Based Complement Alternat Med. (2013) 2013:761454. 10.1155/2013/761454 24058377 PMC3766593

[19] Chang JM Hung LM Chyan YJ Cheng CM Wu RY. Carthamus tinctorius enhances the antitumor activity of dendritic cell vaccines via polarization toward Th1 cytokines and increase of cytotoxic T lymphocytes. Evid Based Complement Alternat Med. (2011) 2011:274858. 10.1093/ecam/nen068 19001481 PMC3096489

[20] Zhao X Lu Y Tao Y Huang Y Wang D Hu Y. Salidroside liposome formulation enhances the activity of dendritic cells and immune responses. Int Immunopharmacol. (2013) 17:1134–40. 10.1016/j.intimp.2013.10.016 24188805

[21] Du X Zhao B Li J Cao X Diao M Feng H. Astragalus polysaccharides enhance immune responses of HBV DNA vaccination via promoting the dendritic cell maturation and suppressing Treg frequency in mice. Int Immunopharmacol. (2012) 14:463–70. 10.1016/j.intimp.2012.09.006 23006659

[22] Lin KY Guarnieri FG Staveley-O'Carroll KF Levitsky HI August JT Pardoll DM. Treatment of established tumors with a novel vaccine that enhances major histocompatibility class II presentation of tumor antigen. Cancer Res. (1996) 56:21–6. 8548765

[23] Song YC Cheng HY Leng CH Chiang SK Lin CW Chong P. A novel emulsion-type adjuvant containing CpG oligodeoxynucleotides enhances CD8^+^ T-cell-mediated anti-tumor immunity. J Control Release. (2014) 173:158–65. 10.1016/j.jconrel.2013.10.027 24177312

[24] Song YC Chou AH Homhuan A Huang MH Chiang SK Shen KY. Presentation of lipopeptide by dendritic cells induces anti-tumor responses via an endocytosis-independent pathway *in vivo*. J Leukoc Biol. (2011) 90:323–32. 10.1189/jlb.0111046 21521754

[25] Song YC Liu SJ. A TLR9 agonist enhances the anti-tumor immunity of peptide and lipopeptide vaccines via different mechanisms. Sci Rep. (2015) 5:12578. 10.1038/srep12578 26215533 PMC4517169

[26] Lutz MB Kukutsch N Ogilvie AL Rossner S Koch F Romani N. An advanced culture method for generating large quantities of highly pure dendritic cells from mouse bone marrow. J Immunol Methods. (1999) 223:77–92. 10.1016/S0022-1759(98)00204-X 10037236

[27] Mauel S Steinbach F Ludwig H. Monocyte-derived dendritic cells from horses differ from dendritic cells of humans and mice. Immunology. (2006) 117:463–73. 10.1111/j.1365-2567.2005.02319.x 16556260 PMC1782256

[28] Chi KH Liu SJ Li CP Kuo HP Wang YS Chao Y. Combination of conformal radiotherapy and intratumoral injection of adoptive dendritic cell immunotherapy in refractory hepatoma. J Immunother. (2005) 28:129–35. 10.1097/01.cji.0000154248.74383.5e 15725956

[29] Kim JJ Hwang YH Kang KY Kim I Kim JB Park JH. Enhanced dendritic cell maturation by the B-chain of Korean mistletoe lectin (KML-B), a novel TLR4 agonist. Int Immunopharmacol. (2014) 21:309–19. 10.1016/j.intimp.2014.05.010 24859056

[30] Li J Wang X Wang W Luo J Aipire A Zhang F. Pleurotus ferulae water extract enhances the maturation and function of murine bone marrow-derived dendritic cells through TLR4 signaling pathway. Vaccine. (2015) 33:1923–33. 10.1016/j.vaccine.2015.02.063 25748337

[31] Song D He Z Wang C Yuan F Dong P Zhang W. Regulation of the exopolysaccharide from an anamorph of Cordyceps sinensis on dendritic cell sarcoma (DCS) cell line. Eur J Nutr. (2013) 52:687–94. 10.1007/s00394-012-0373-x 22610670

[32] Xiao G Miyazato A Abe Y Zhang T Nakamura K Inden K. Activation of myeloid dendritic cells by deoxynucleic acids from Cordyceps sinensis via a Toll-like receptor 9-dependent pathway. Cell Immunol. (2010) 263:241–50. 10.1016/j.cellimm.2010.04.006 20451901

[33] Zhang S Nie S Huang D Huang J Wang Y Xie M. Polysaccharide from Ganoderma atrum evokes antitumor activity via Toll-like receptor 4-mediated NF-kappaB and mitogen-activated protein kinase signaling pathways. J Agric Food Chem. (2013) 61:3676–82. 10.1021/jf4004225 23514335

[34] Reis e Sousa C Dendritic cells in a mature age. Nat Rev Immunol. (2006) 6:476–83. 10.1038/nri1845 16691244

[35] Guo Q Li J Lin H. Effect and molecular mechanisms of traditional chinese medicine on regulating tumor immunosuppressive microenvironment. Biomed Res Int. (2015) 2015:261620. 10.1155/2015/261620 26161392 PMC4486742

[36] Ye JH Liu MH Zhang XL He JY. Chemical profiles and protective effect of hedyotis diffusa willd in lipopolysaccharide-induced renal inflammation mice. Int J Mol Sci. (2015) 16:27252–69. 10.3390/ijms161126021 26580602 PMC4661879

[37] De la Fuente M Medina S Baeza I Jimenez L. Improvement of leucocyte functions in mature and old mice after 15 and 30 weeks of diet supplementation with polyphenol-rich biscuits. Eur J Nutr. (2011) 50:563–73. 10.1007/s00394-010-0163-2 21221978

[38] He ZW Wei W Li SP Ling Q Liao KJ Wang X. Anti-allodynic effects of obtusifolin and gluco-obtusifolin against inflammatory and neuropathic pain. Biol Pharm Bull. (2014) 37:1606–16. 10.1248/bpb.c14-00307 25070277

[39] Katayama S Ohno F Mitani T Akiyama H Nakamura S. Rutinosylated ferulic acid attenuates food allergic response and colitis by upregulating regulatory T cells in mouse models. J Agric Food Chem. (2017) 65:10730–37. 10.1021/acs.jafc.7b03933 29141406

[40] Lin JP Yang JS Lu CC Chiang JH Wu CL Lin JJ. Rutin inhibits the proliferation of murine leukemia WEHI-3 cells *in vivo* and promotes immune response *in vivo*. Leuk Res. (2009) 33:823–8. 10.1016/j.leukres.2008.09.032 19010542

[41] Pragasam SJ Venkatesan V Rasool M. Immunomodulatory and anti-inflammatory effect of p-coumaric acid, a common dietary polyphenol on experimental inflammation in rats. Inflammation. (2013) 36:169–76. 10.1007/s10753-012-9532-8 22923003

[42] Deng WP Chao MW Lai WF Sun C Chung CY Wu CC. Correction of malignant behavior of tumor cells by traditional Chinese herb medicine through a restoration of p53. Cancer Lett. (2006) 233:315–27. 10.1016/j.canlet.2005.03.031 15882924

[43] Munagala R Aqil F Jeyabalan J Gupta RC. Tanshinone IIA inhibits viral oncogene expression leading to apoptosis and inhibition of cervical cancer. Cancer Lett. (2015) 356:536–46. 10.1016/j.canlet.2014.09.037 25304375

[44] Zheng J Deng YP Lin C Fu M Xiao PG Wu M. Arsenic trioxide induces apoptosis of HPV16 DNA-immortalized human cervical epithelial cells and selectively inhibits viral gene expression. Int J Cancer. (1999) 82:286–92. 10.1002/(sici)1097-0215(19990719)82:2<286::aid-ijc21>3.0.co;2-k 10389765

[45] Xu YX Yuan L Improvement of cervical microenvironment and effect of Erhuang Powder on CIN I accompanied by human papillomavirus infection according to Th1/Th2 immune balance. Liaoning J Tradit Chin Med. (2016) 43:962–65. Available online at: https://caod.oriprobe.com/articles/48266301/Improvement_of_Cervical_Microenvironment_and_Effec.htm

[46] Ben Sghaier M Pagano A Mousslim M Ammari Y Kovacic H Luis J. Rutin inhibits proliferation, attenuates superoxide production and decreases adhesion and migration of human cancerous cells. Biomed Pharmacother. (2016) 84:1972–78. 10.1016/j.biopha.2016.11.001 27829548

[47] Gautam R Singh M Gautam S Rawat JK Saraf SA Kaithwas G. Rutin attenuates intestinal toxicity induced by Methotrexate linked with anti-oxidative and anti-inflammatory effects. BMC Complement Altern Med. (2016) 16:99. 10.1186/s12906-016-1069-1 26965456 PMC4785621

[48] Sharma S Ali A Ali J Sahni JK Baboota S. Rutin: therapeutic potential and recent advances in drug delivery. Expert Opin Investig Drugs. (2013) 22:1063–79. 10.1517/13543784.2013.805744 23795677

[49] Chen Y Lin Y Li Y Li C. Total flavonoids of Hedyotis diffusa Willd inhibit inflammatory responses in LPS-activated macrophages via suppression of the NF-kappaB and MAPK signaling pathways. Exp Ther Med. (2016) 11:1116–22. 10.3892/etm.2015.2963 26998046 PMC4774565

[50] Dong Q Ling B Gao B Maley J Sammynaiken R Yang J. Hedyotis diffusa water extract diminished the cytotoxic effects of chemotherapy drugs against human breast cancer MCF7 cells. Nat Prod Commun. (2014) 9:699–700. 10.1177/1934578X1400900529 25026725

[51] Kim Y Park EJ Kim J Kim Y Kim SR Kim YY. Neuroprotective constituents from Hedyotis diffusa. J Nat Prod. (2001) 64:75–8. 10.1021/np000327d 11170670

[52] Lu CM Yang JJ Wang PY Lin CC. A new acylated flavonol glycoside and antioxidant effects of Hedyotis diffusa. Planta Med. (2000) 66:374–7. 10.1055/s-2000-8544 10865461

[53] Topalian SL Drake CG Pardoll DM. Targeting the PD-1/B7-H1(PD-L1) pathway to activate anti-tumor immunity. Curr Opin Immunol. (2012) 24:207–12. 10.1016/j.coi.2011.12.009 22236695 PMC3319479

