# Supplementary figures and images for: A Potential Herbal Adjuvant Combined With a Peptide-Based Vaccine Acts Against HPV-Related Tumors Through Enhancing Effector and Memory T-Cell Immune Responses
Source: Front Immunol. 2020 Feb 20;11:62. doi: 10.3389/fimmu.2020.00062 (PMC7044417; doi:10.3389/fimmu.2020.00062)

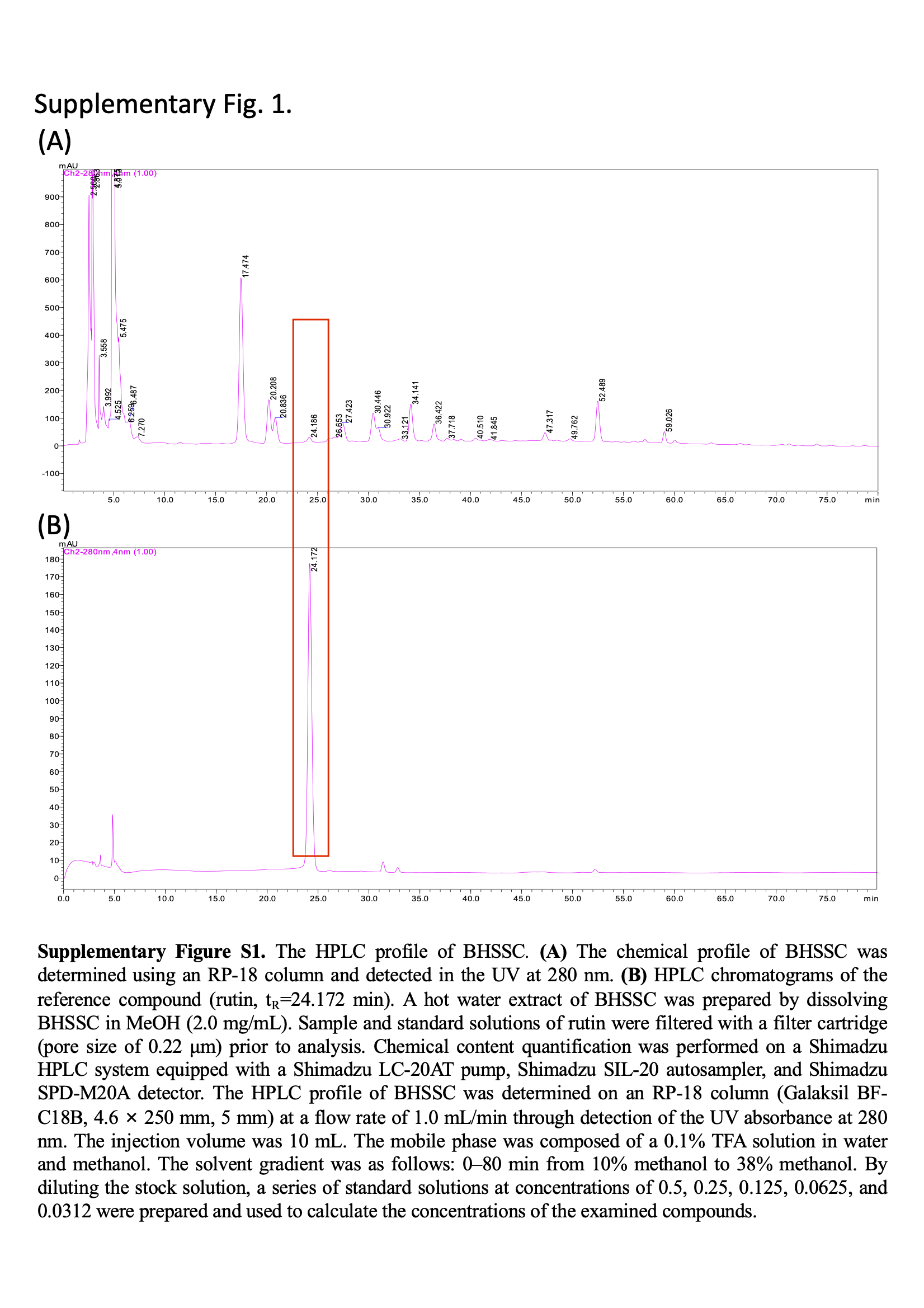

Supplement: Supplementary file 1 [file Image_1.TIFF]

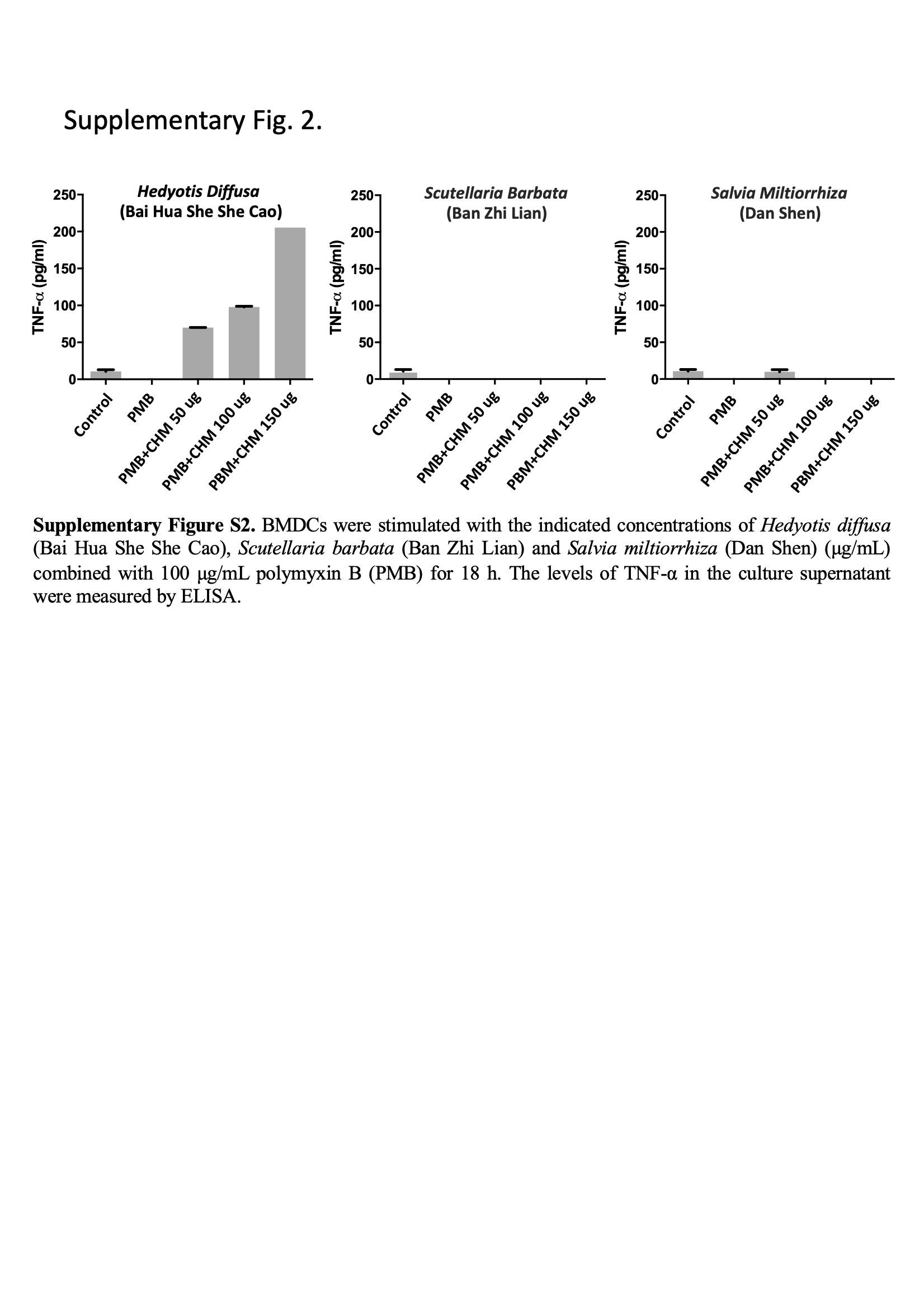

Supplement: Supplementary file 2 [file Image_2.TIFF]

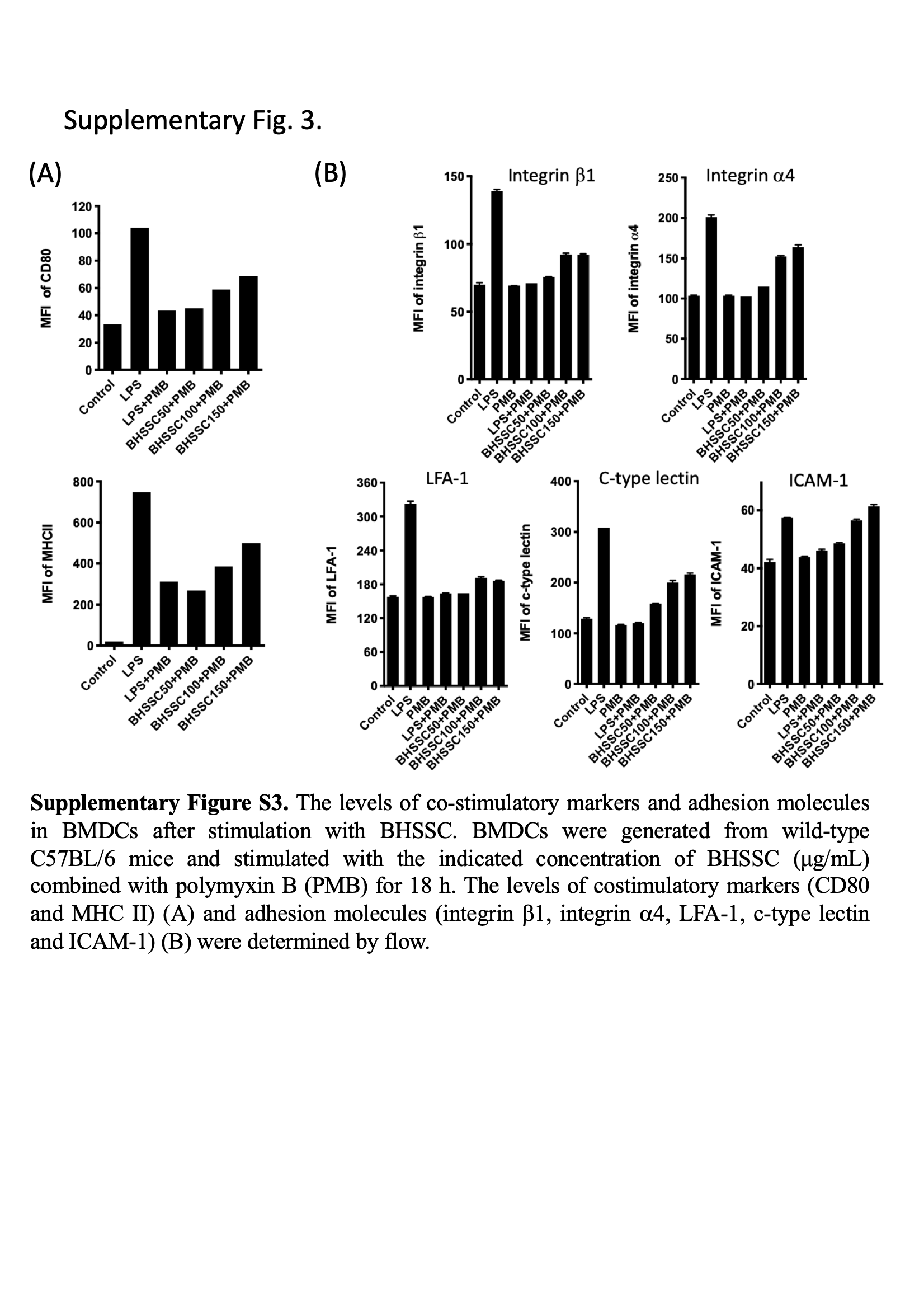

Supplement: Supplementary file 3 [file Image_3.TIFF]

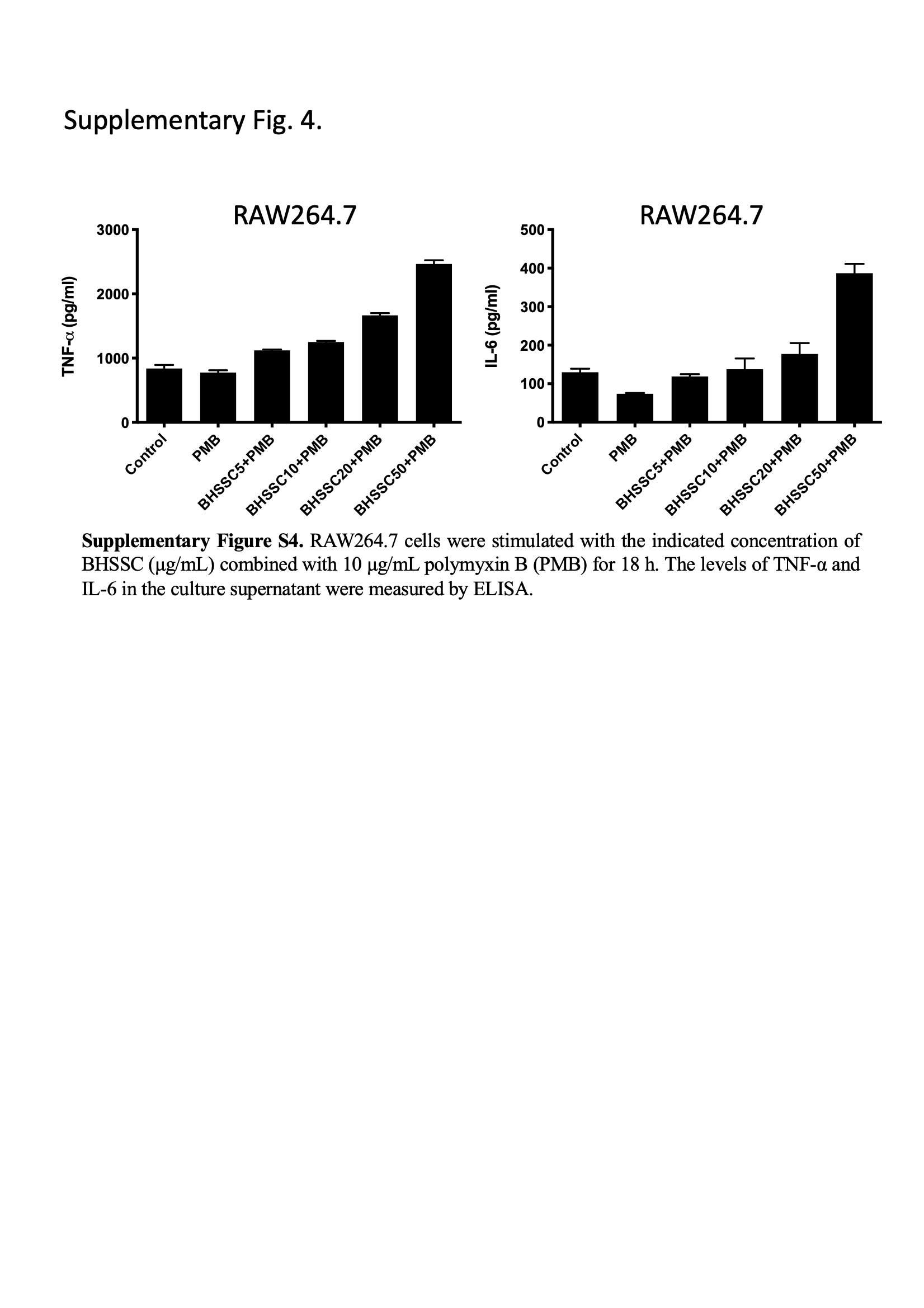

Supplement: Supplementary file 4 [file Image_4.TIFF]

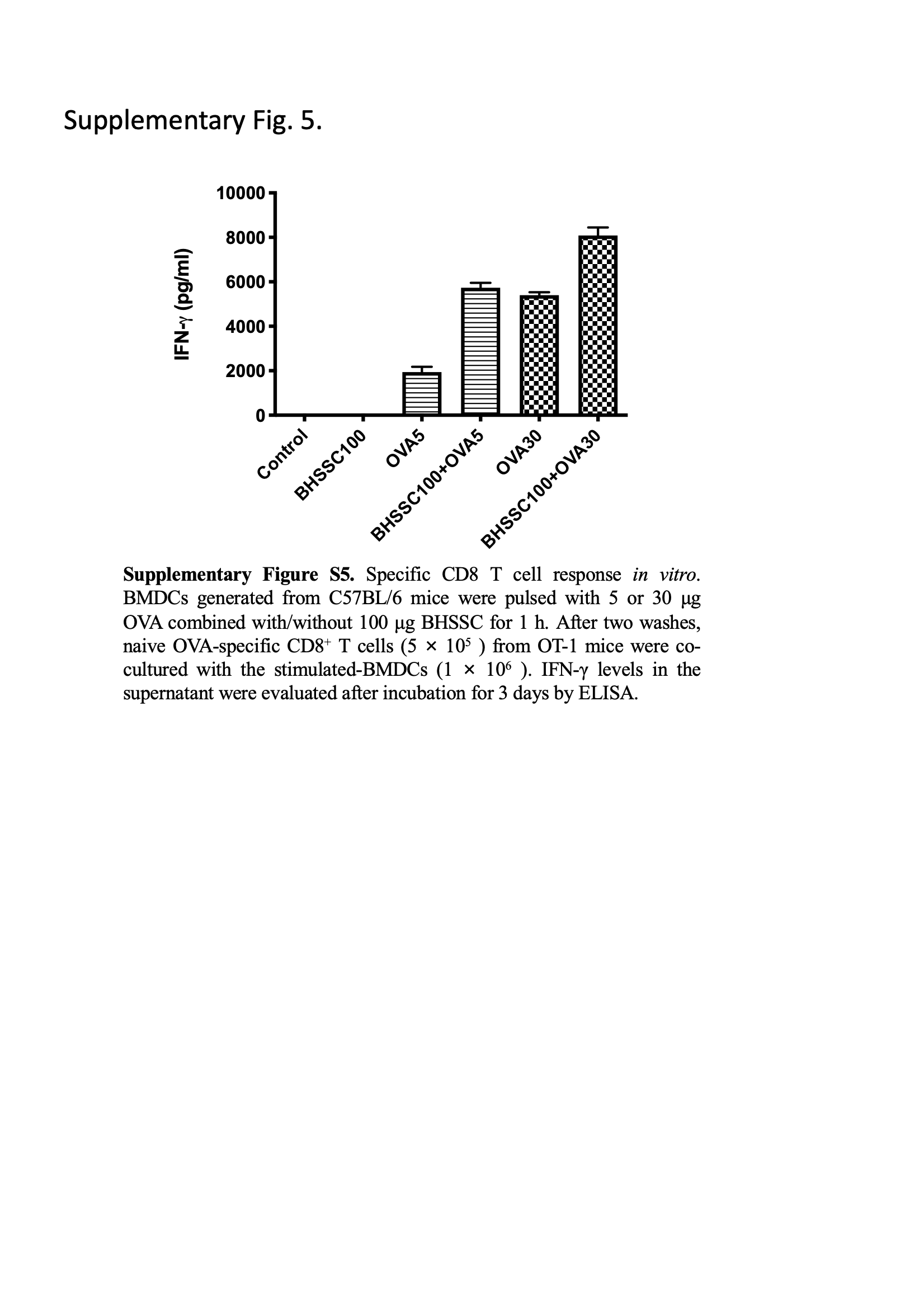

Supplement: Supplementary file 5 [file Image_5.TIFF]

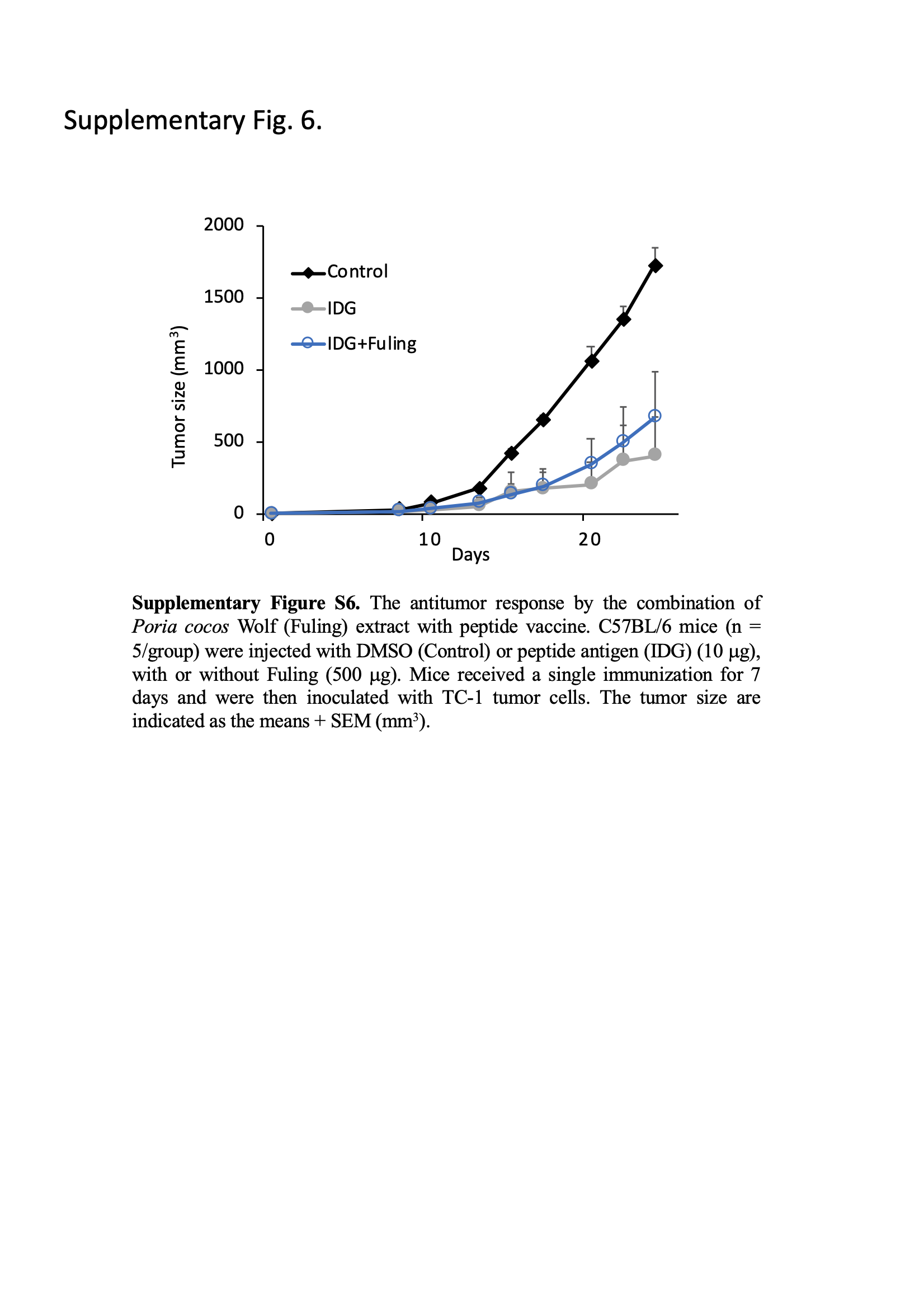

Supplement: Supplementary file 6 [file Image_6.TIFF]

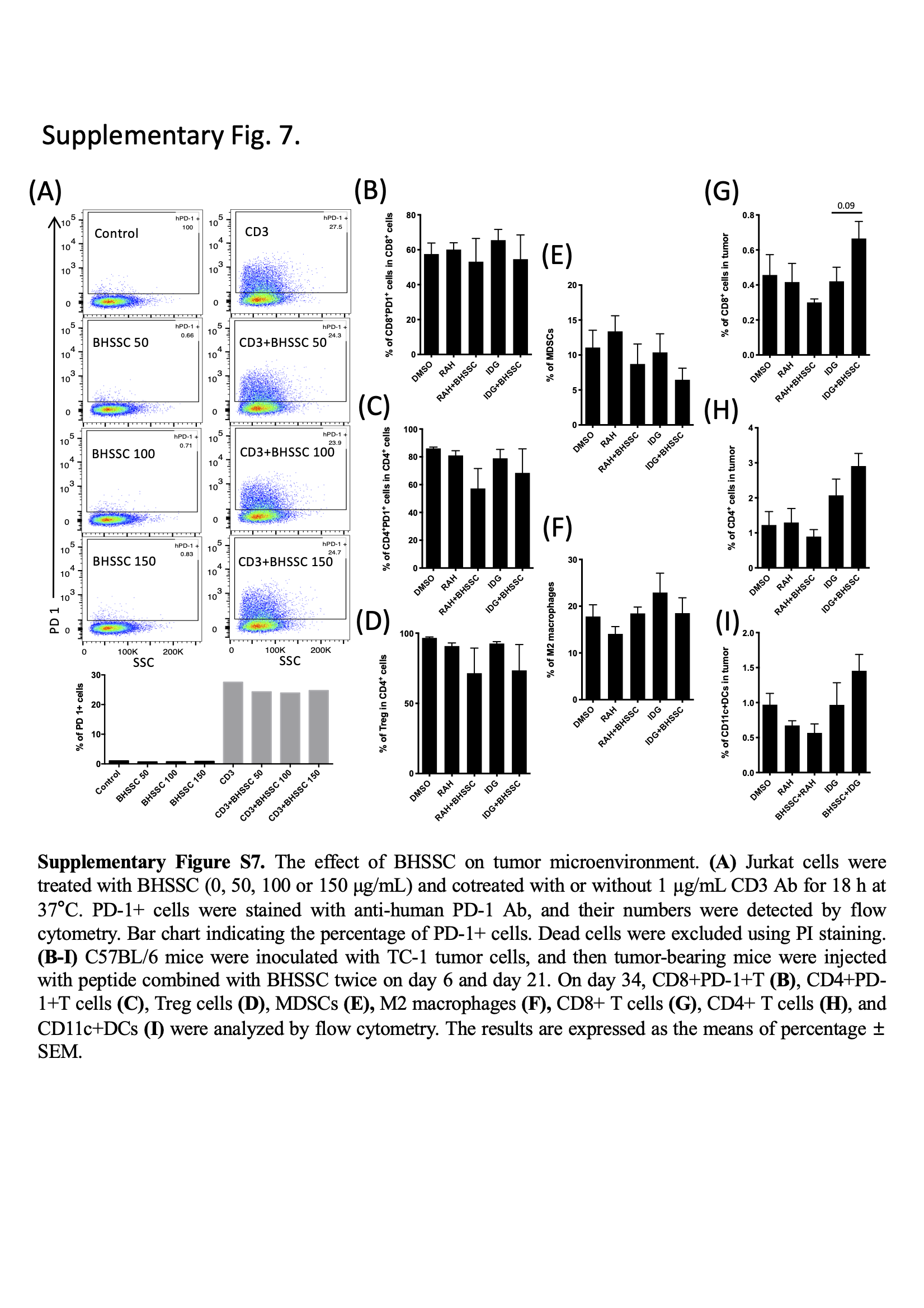

Supplement: Supplementary file 7 [file Image_7.TIFF]

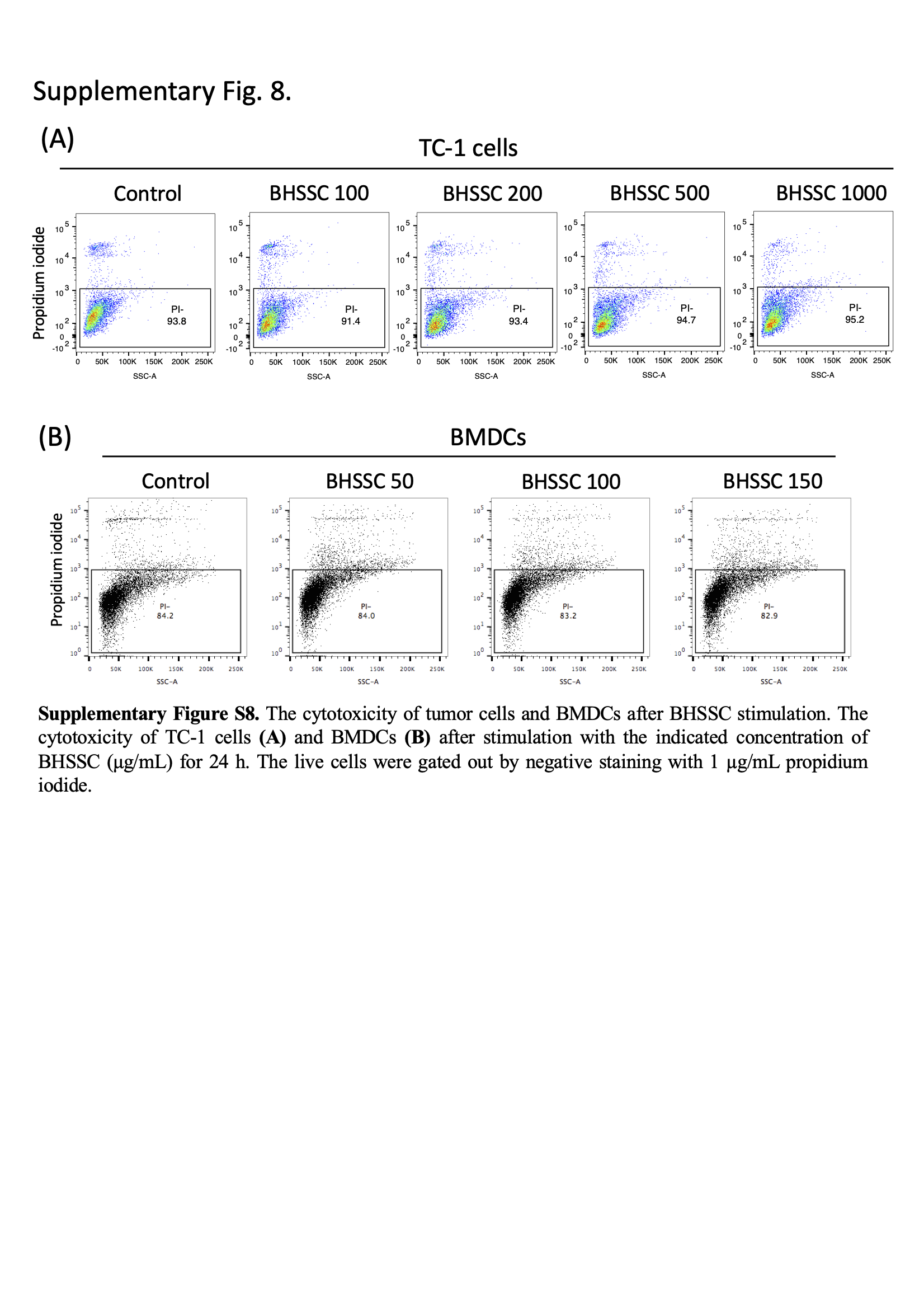

Supplement: Supplementary file 8 [file Image_8.TIFF]

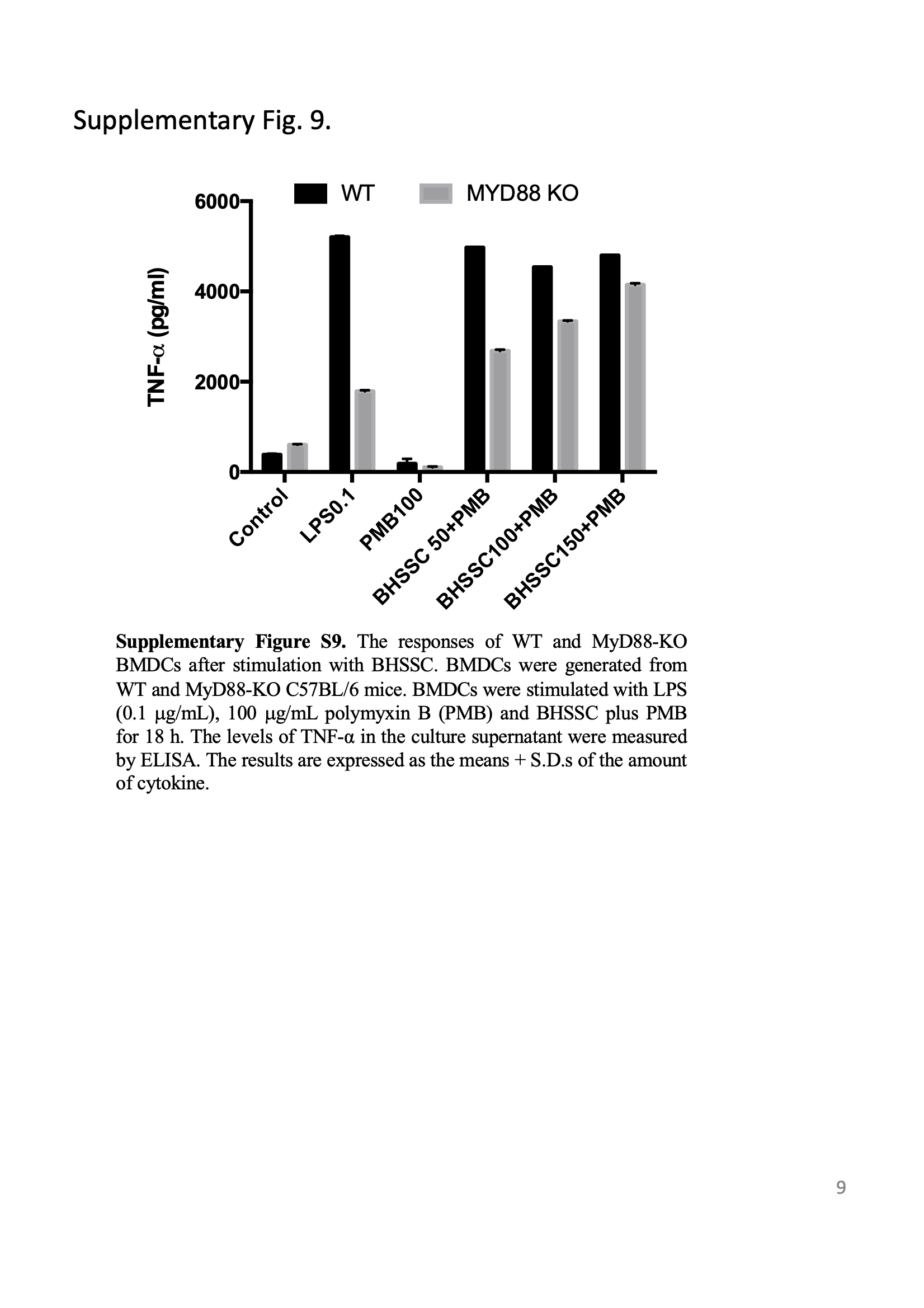

Supplement: Supplementary file 9 [file Image_9.TIFF]

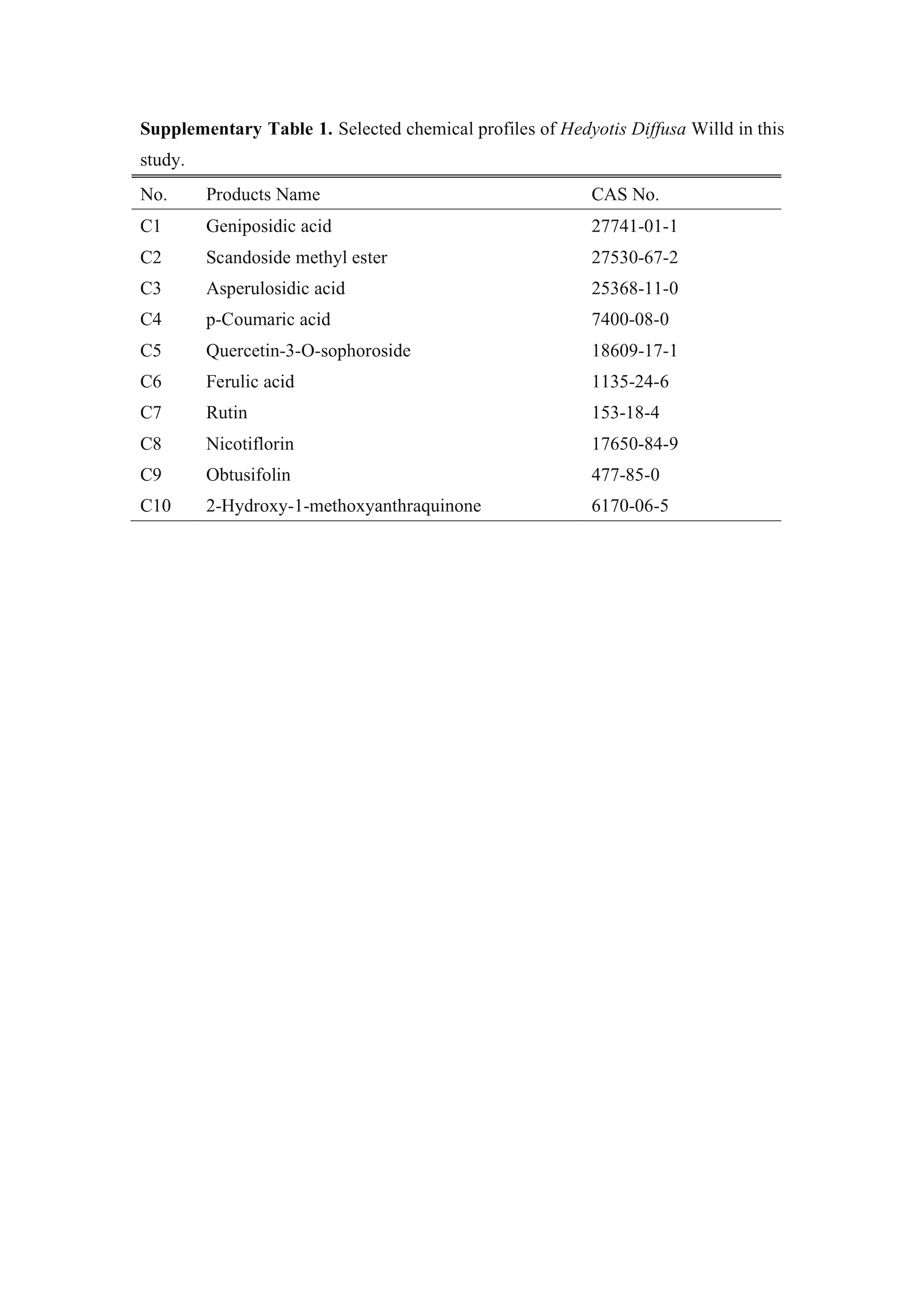

Supplement: Supplementary file 10 [file Image_10.TIFF]
